# Benchmarking brain organoid recapitulation of fetal corticogenesis

**DOI:** 10.1038/s41398-022-02279-0

**Published:** 2022-12-20

**Authors:** Cristina Cheroni, Sebastiano Trattaro, Nicolò Caporale, Alejandro López-Tobón, Erika Tenderini, Sara Sebastiani, Flavia Troglio, Michele Gabriele, Raul Bardini Bressan, Steven M. Pollard, William T. Gibson, Giuseppe Testa

**Affiliations:** 1 Department of Experimental Oncology, European Institute of Oncology IRCCS, Milan, Italy; 2 Department of Oncology and Hemato-Oncology, University of Milan, Via Santa Sofia 9, 20122 Milan, Italy; 3 Human Technopole, Viale Rita Levi-Montalcini 1, 20157 Milan, Italy; 4 Centre for Regenerative Medicine, Institute for Regeneration and Repair, and Edinburgh Cancer Research UK Centre, University of Edinburgh, Edinburgh, EH16 4UU UK; 5 BC Children’s Hospital Research Institute, 950 West 28th Avenue, Vancouver, BC V5Z 4H4 Canada; 6 Department of Medical Genetics, University of British Columbia, Vancouver, BC V6T 1Z4 Canada; 7 Present Address: Herophilus, Inc., San Francisco, CA 94107 USA; 8 Present Address: Telethon Institute of Genetics and Medicine (TIGEM), Pozzuoli, Via Campi Flegrei, 34 - 80078 Naples, Italy; 9 Present Address: Department of Molecular Neurobiology, Donders Institute for Brain, Cognition and Behavior, Radboud University Nijmegen, Nijmegen, The Netherlands; 10 Present Address: Department of Biological Engineering, Massachusetts Institute of Technology, Cambridge, MA 02139 USA; 11 Present Address: The Broad Institute of MIT and Harvard, Cambridge, MA 02139 USA; 12 Present Address: Koch Institute for Integrative Cancer Research, Cambridge, MA 02139 USA

**Keywords:** Molecular neuroscience, Stem cells, Psychiatric disorders

## Abstract

Brain organoids are becoming increasingly relevant to dissect the molecular mechanisms underlying psychiatric and neurological conditions. The in vitro recapitulation of key features of human brain development affords the unique opportunity of investigating the developmental antecedents of neuropsychiatric conditions in the context of the actual patients’ genetic backgrounds. Specifically, multiple strategies of brain organoid (BO) differentiation have enabled the investigation of human cerebral corticogenesis in vitro with increasing accuracy. However, the field lacks a systematic investigation of how closely the gene co-expression patterns seen in cultured BO from different protocols match those observed in fetal cortex, a paramount information for ensuring the sensitivity and accuracy of modeling disease trajectories. Here we benchmark BO against fetal corticogenesis by integrating transcriptomes from in-house differentiated cortical BO (CBO), other BO systems, human fetal brain samples processed in-house, and prenatal cortices from the BrainSpan Atlas. We identified co-expression patterns and prioritized hubs of human corticogenesis and CBO differentiation, highlighting both well-preserved and discordant trends across BO protocols. We evaluated the relevance of identified gene modules for neurodevelopmental disorders and psychiatric conditions finding significant enrichment of disease risk genes especially in modules related to neuronal maturation and synapsis development. The longitudinal transcriptomic analysis of CBO revealed a two-step differentiation composed of a fast-evolving phase, corresponding to the appearance of the main cell populations of the cortex, followed by a slow-evolving one characterized by milder transcriptional changes. Finally, we observed heterochronicity of differentiation across BO models compared to fetal cortex. Our approach provides a framework to directly compare the extent of in vivo/in vitro alignment of neurodevelopmentally relevant processes and their attending temporalities, structured as a resource to query for modeling human corticogenesis and the neuropsychiatric outcomes of its alterations.

## Introduction

The introduction of cell reprogramming technologies and 3D brain organoids (BO) have made the spatial and temporal dynamics of human brain development experimentally accessible. BO are thus becoming central to the investigation of how genetic vulnerabilities or environmental perturbations can alter physiological neurodevelopment and seed the unfolding of psychiatric and neurological conditions [1, 2]. As we and others recently demonstrated, the exposure of specific windows of vulnerability is proving of particular value, highlighting how temporally defined or even transient alterations in neurodevelopmental trajectories can bring about major mental health outcomes, from language delay to autism spectrum disorders (ASD) [3–5]. Indeed, a growing body of literature testifies to the edge that BO are bringing to the modeling of complex neuropsychiatric disorders, from ASD to schizophrenia, bipolar disorder, and beyond [6–8]. Given the central role of the cortex for the higher-order functions primarily affected in neuropsychiatric conditions [9], determining how closely BO recapitulate human cerebral corticogenesis is thus crucial for harnessing their full potential as in vitro models of neurodevelopmental processes and their physiopathological outcomes. To this end, comparison between ex vivo human fetal cortex and BO gene expression has started to uncover the extent of this recapitulation, alongside the peculiarities of different methods. Specifically, the transcriptomic and epigenomic landscapes of BO were characterized and compared to isogenic fetal cortices, finding significant overlaps [10], as well as to mice data, highlighting human-specific genes involved in lineage establishment [11]. Very long-term BO cultures were meanwhile shown to capture early post-natal developmental transitions [12], while spatial similarity maps of BO against reference were generated to assess organoid engineering protocols and to annotate cell fates [13]. Further detail emerged from integrative analyses showing an overexpression of extracellular matrix (ECM)-related genes in BO associated with the first steps of differentiation in 2D [14], a higher vulnerability to cellular stress in some organoid cultures compared to primary tissue [15, 16], and the existence of protocol-specific transcriptional bypasses in BO differentiation [17].

These efforts have provided a wealth of information, which remains, however, difficult to harmonize due to the lack of dedicated resources where transcriptional hubs of development/differentiation are categorized, ranked, and made available for consultation. Such tools are needed to help researchers select protocols and time-points based not only on the expression of genes of interest but also on the broader context of functional partitions and temporal dynamics of that expression. Building on these considerations, we benchmarked selected BO paradigms against human corticogenesis with the aim of templating such a resource through a framework that allows its adaptation and growth as the neural modeling field continues to mature. Specifically, we profiled in-house a cohort of cortical BO (CBO) [18, 19], derived from multiple individuals and differentiation rounds over 200 days, and integrated it with (i) our in-house cohort of primary samples, (ii) publicly available transcriptomic data from BO different for degree of guidance, and (iii) prenatal cortical samples of the BrainSpan Atlas (BS). The characterization of the gene expression landscape of BS and CBO led to the definition of co-expression patterns relevant for human prenatal corticogenesis and CBO differentiation, as well as to the ranking and categorization in functional domains of their transcriptional hubs. We then cross-compared primary samples, CBO and other BO systems [14, 20, 21]. These analyses revealed the overlap between co-expression patterns of prenatal cortex and CBO, allowed their visualization in the selected BO systems, and pointed at partial heterochronicity in the transcriptional recapitulation of corticogenesis by different BO methods.

In sum, our analyses represent a benchmark for the interrogation of the transcriptional networks characterizing human prenatal corticogenesis and CBO differentiation and uncover their modulation also in other BO protocols of widespread use for neuropsychiatric disease modeling. By comparing co-expression patterns of human corticogenesis and BO differentiation across protocols, our work represents a template for the benchmarking of BO and their applications in disease modeling.

## Results

### Gene co-expression analysis highlights the transcriptional programs of the prenatal human corticogenesis

To categorize the transcriptional hubs of the developing human cortex, we took advantage of the BrainSpan Atlas (BS), the most comprehensive transcriptional characterization of the human brain encompassing both fetal and post-natal/adult stages.

**Fig. 1:**
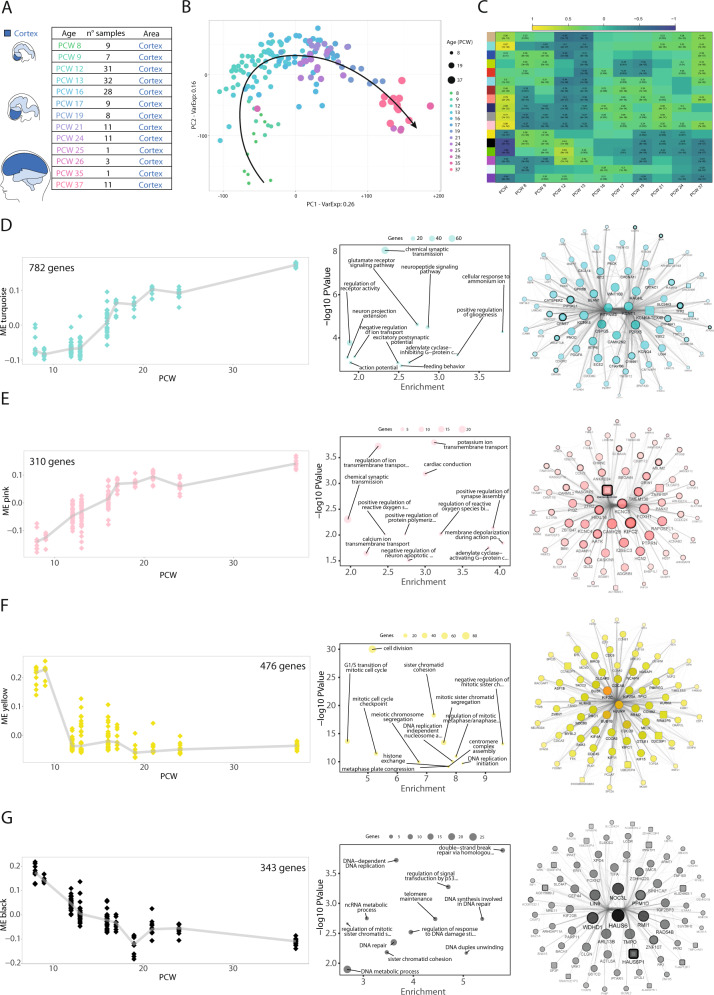
Reconstruction of transcriptional programs of the developing fetal cortex. **A** Cohort of fetal cortical samples from the BrainSpan (BS) Atlas. The number of specimens for each post-conceptional week (PCW) is reported, for a total of 162 bulk RNASeq data points. **B** PCA on prenatal cortical samples from BS. Dot color and size is set according to developmental stage. **C** WGCNA pinpoints gene modules in the developing cortex. The heatmap shows the correlation between the first principal component of each module (module eigengene, ME) and the developmental stage, either as a continuous variable (PCW) or as a categorical variable for each stage. Coefficients of correlation were calculated using Spearman correlation; *P*-values are reported for significant correlations (*P*-value < 0.01). Each row represents a gene module and it is identified with a specific color. **D**–**G** Characterization of BS_Turquoise, BS_Pink, BS_Yellow, and BS_Black modules. For each module, ribbon plots visualize the behavior of the ME (Y-axis) through developmental stages; each dot represents a data point, while the line connects the median value for each PCW. The bubble plots show the *P*-value (Y-axis) and enrichment score (X-axis) for the top-12 GO categories (ranked according to *P*-value, Biological Process domain of the ontology); dot size represents the number of module genes belonging to the GO term (complete results are reported in Supplementary Table 3). Network reconstruction for the top-75 genes of each module, selected according to the intramodular connectivity. Degree, closeness, betweenness, and eigenvector are represented by node label transparency, node color darkness, node border width, and node size/node label font size, respectively. Node shape represents gene biotype, with protein-coding genes as circles and non-coding genes as squares.

In a landmark effort, Miller et al. profiled by microarray fetal brain at mid-gestation [22], establishing a first reference transcriptional atlas. More recently, a multi-modal characterization of human brain was performed [23]. We applied a similar approach at the transcriptome level, focusing our effort specifically on the prenatal cerebral cortex. This allowed us to reconstruct the transcriptional circuitries of the human fetal corticogenesis with increased resolution. We selected a total of 162 data points (Fig. 1A) from cerebral cortex at post-conceptional weeks (PCW) 8–37.

Principal component analysis (PCA) identified developmental stage as the main driver of sample differences, with PC2 separating very early stages (PCW 8–9) from early ones (PCW 12–13), followed by a progression of time-points in PC1 (Fig. 1B). Likewise, stage-wise correlation analysis detected a first shift in the transcriptional landscape from PCW 8–9 to 12–24 and a second, less clear-cut, characterizing PCW 25–37 (Supplementary Fig. 1A). The same analysis on BS post-natal cortical samples showed higher homogeneity compared to prenatal time-points (Supplementary Fig. 1B), indicating a more profound transcriptional evolution during the fetal phase, especially till PCW24.

We applied gene ontology enrichment analysis (GO) to the top 600 genes associated with PC1 or PC2 according to PCA loadings. We retrieved for PC1 categories associated with ion channels, lipid metabolism, and transcriptional regulation, while we found terms related to neuronal maturation and cell division for PC2 (Fig. 1B; Supplementary Fig. 1C, D).

To identify the transcriptional programs regulating corticogenesis in an unsupervised manner, we applied weighted gene co-expression network analysis (WGCNA), uncovering 17 gene co-expression modules (Fig. 1C, Supplementary Fig. 2A–C and Supplementary File 1). We summarized the co-expression profile of each module by its first PC (module eigengene, ME) and related it to developmental stage, highlighting several modules as positively or negatively correlated. BS_Turquoise, BS_Pink, BS_Grey60, and BS_Midnightblue showed positive correlation, while BS_Black and BS_Green were negatively associated with developmental progression (Fig. 1C). Other modules displayed changes in more restricted time windows (e.g. BS_Yellow, BS_Blue, BS_Red, Fig. 1C). The functional characterization of BS modules pointed to clear-cut biological domains for several of them, such as glutamatergic transmission and synapse for BS_Turquoise, ion channels for BS_Pink, DNA replication for BS_Black, and cell division for BS_Yellow (Fig. 1D–G). Finally, we reconstructed the co-expression network selecting the top-75 genes (according to intramodular connectivity) and then applied network analysis. Central nodes of BS_Turquoise and BS_Pink networks were related to neuronal functions and included synaptic proteins (CAMK2N2, CAMK2B, and GDA), receptor subunits (GRIN1, GRIN3A) and potassium channel subunits (KCNT1, KCNQ4, KCNC3, KCNC4) (Fig. 1D–G). BS_Midnight-blue included the upper layer neuron markers CUX2 and SATB2 among its most central genes (Supplementary Fig. 2D). BS_Yellow and BS_Black hubs were strongly enriched in cell cycle genes.

The reconstructed networks also encompassed genes less studied in corticogenesis (e.g. for BS_Turquoise CATSPERZ, HAGHL, ADAMTS8, and KCN4-TEX40), thus allowing us to hypothesize their involvement by a guilt-by-association approach (Fig. 1D–G and Supplementary Fig. 2D).

Overall, we identified transcriptional circuitries related to developmental transitions and functional domains of human corticogenesis, reconstructing relevant gene co-expression networks. Main modules, their behavior, and the relevance of every gene composing them are available in Supplementary File 1. To our knowledge, this collection represents the first resource of gene co-expression networks specifically focusing on prenatal corticogenesis that can be used to explore genes of interest in relation to physiological or pathological conditions.

### Cortical brain organoids globally resemble the developing human fetal cortex and evolve in two steps

Upon characterization of the transcriptional dynamics defining human corticogenesis, we investigated the extent of their recapitulation in BO, with an experimental design that allowed us to measure interindividual and technical variability.

CBO has been shown to recapitulate key events of human corticogenesis [5, 18, 19]. Indeed, immunohistochemical characterization confirmed the presence of key population markers for neural stem cells (SOX2), cell cycle (KI67), apical progenitors (PAX6), intermediate progenitors (TBR2), outer radial glia (HOPX), layer-specific neuronal markers (BCL11B, SATB2), and astrocytes (GFAP) (Supplementary Fig. 3 and 4 and [5, 18]).

**Fig. 2:**
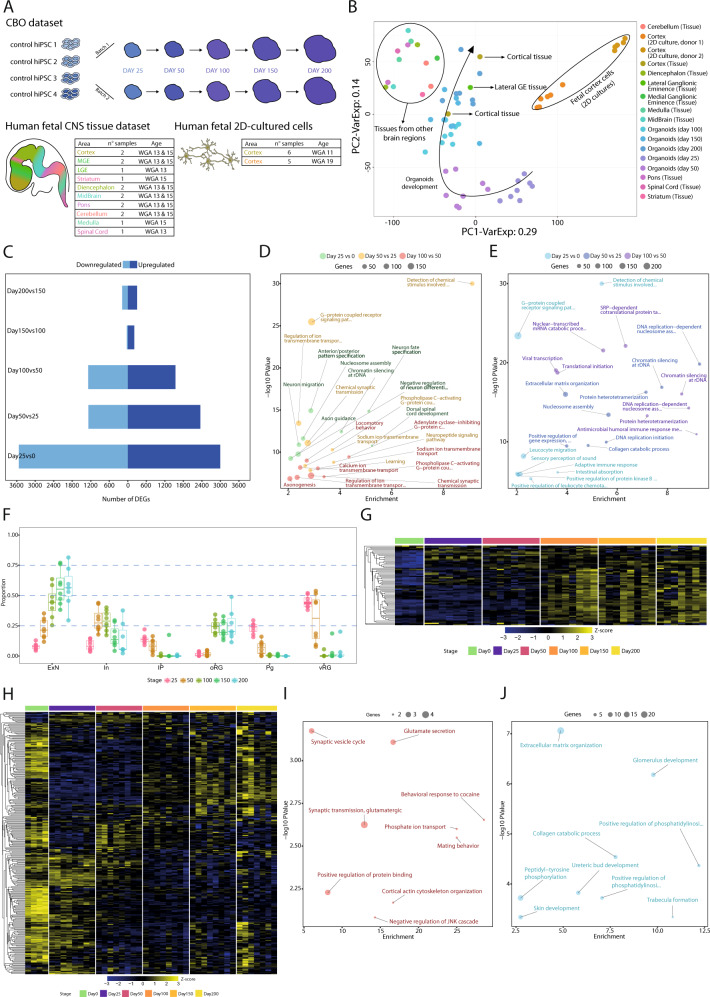
Principal component and differential expression analyses uncovered CBO temporal dynamics. **A** Experimental design, detailing the number of individuals, batches and time-points for CBO and number of samples, brain area and WGA for fetal samples. **B** PCA on CBO, fetal brain tissues and 2D cultured fetal cortical progenitors, distinguished by dot color. The arrow highlights the distribution of CBO samples throughout stage progression. **C** Number of DEGs (FDR < 0.05, log2FC > 1 as absolute value) from stagewise differential expression analysis in the CBO dataset. Barplots show the number of upregulated and downregulated genes for each comparison. **D**, **E** Bubble plots showing the functional characterization of upregulated (**D**) and downregulated (**E**) genes by functional enrichment analysis for the following comparisons: (I) Day25 vs Day0; (II) Day50 vs Day25; (III) Day100 vs Day50. The top-8 GO categories from the Biological Process domain of the GO are reported for each comparison (details are reported in Supplementary Table 3). **F** Boxplots displaying the estimated proportion of excitatory neurons (ExN), inhibitory neurons (In), intermediate progenitors (IP), outer radial glia (oRG), cycling progenitors (Pg), and ventricular radial glia (vRG) from bulk deconvolution in each stage of CBO differentiation. **G**, **H** Heatmaps showing the behavior of cortex-specific upregulated (**G**) and downregulated (**H**) DEGs in the CBO dataset. Values are shown as Z-scores calculated on the expression values. **I**, **J** Bubble plots showing the functional characterization of the genes in (**G**) and (**H**), respectively.

We analyzed the transcriptional landscape of in-house differentiated CBO generated as previously described [18], for a total of 39 single-organoid and 4 hiPSCs samples from 4 control lines profiled over 200 days. We profiled single organoids to tackle technical variability of differentiation and we analyzed 2 independent organoid batches to measure reproducibility. In addition, we profiled a cohort of primary fetal CNS tissues (weeks of gestational age -WGA- 13 and 15) and 2D cultures (WGA 11, donor 1, and 19, donor 2) for a total of 4 individuals (described in [5], Fig. 2A). This dataset, having been processed as CBO in terms of sample preparation, library generation, sequencing platform, and computational pipelines, allowed a direct comparisons between the fetal tissue and the organoid model.

PCA showed temporal evolution of CBO with early stages forming individual clusters and later time-points resulting more intermingled. CBO evolved towards the fetal tissue, with clustering of mature organoids in proximity of fetal cortex. Conversely, 2D cultures clustered apart (Fig. 2B). Specific focus on CBO and relative functional analysis allowed us to associate PC1-driving genes to cell cycle and neuronal differentiation (Supplementary Fig. 5A–C).

To quantitatively characterize CBO evolution, we performed stage-wise differential expression analysis (DEA) comparing each stage against the previous. This approach highlighted a biphasic differentiation dynamic, with fast changes until day 100 followed by subtler modulations (Fig. 2C). The fast-evolving phase was characterized by a large number of differentially expressed genes (DEGs), related to neuronal fate commitment and maturation (upregulated) and cell cycle, transcription, and translation dynamics (downregulated) (Fig. 2D, E). The number of DEGs decreased considerably in the slow-evolving phase and related functional categories were generally less clear-cut (Supplementary Fig. 5D, E) [24–30].

We then analyzed dynamic changes among DEGs by visualizing the fold-change of consecutive comparisons (e.g. day 50vs25 against 100vs50, etc). Neuronal differentiation regulators, such as EOMES, LHX2, and FEZF2 [31–33], were modulated dynamically in the fast-evolving phase, while we detected upregulation of the astrocytic markers HEPACAM, AQP4, AGT, and APOE in the slow-evolving phase [34–37]. We also observed upregulation of GABAergic interneuron markers after day 100, in line with other studies [38, 39] (Supplementary Fig. 5F).

We then estimated CBO cell-type proportions with bulk deconvolution exploiting a scRNAseq atlas of the developing human cortex as reference [40]. Deconvolution methods have already been employed to estimate cell type proportions in the fetal brain [23] and have been shown to give reliable estimates when benchmarked against immunohistochemical quantification of the main adult brain cell types, overcoming some of the selection biases affecting single-cell estimations [41]. From day 25 till day 100 CBO showed increase of excitatory neurons mirrored by a drop in ventricular radial glia, cycling progenitors, and intermediate progenitors. Outer radial glia increased from day 100 onwards (Fig. 2F).

Lastly, we identified cortex-enriched genes by comparing the cortical samples of our in-house fetal brain tissue dataset (WGA 13, 15) against hiPSCs and subsequently excluding common DEGs found with the same approach for other brain areas. We analyzed expression of cortex-enriched DEGs in CBO and found upregulation of genes related to glutamatergic neuron function alongside downregulation of ECM genes (Fig. 2G–J). In sum, we determined the transcriptional dynamics of CBO differentiation and we found that CBO resembled the transcriptome of human fetal cortex in terms of key cell populations and transcriptional programs, with a period of vast transcriptional changes followed by a plateau after day 100 in culture. This pattern of differentiation is in line with what has already been reported previously in BO [14] and reflects the shift from a first phase with predominance of progenitors to a second in which maturing neurons become the predominant population, which occurs within 100 days of differentiation for CBO.

### WGCNA identifies cortical brain organoids’ differentiation trajectories towards the glutamatergic fate

**Fig. 3:**
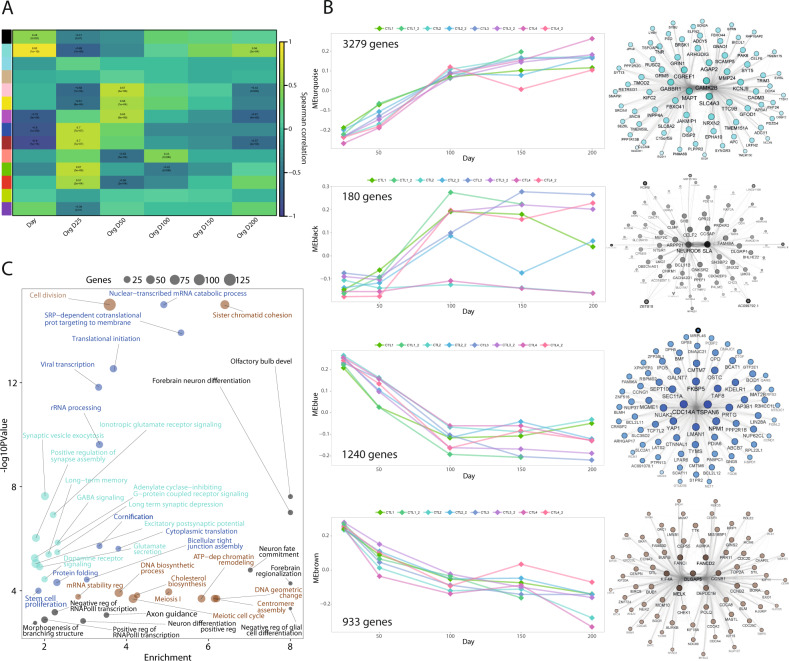
Cortical brain organoids evolve towards the glutamatergic fate. **A** Heatmap showing the correlation between gene modules (summarized as ME) identified by WGCNA in cortical brain organoid (CBO) dataset and differentiation stage either as a continuous variable (Day) or a categorical variable for each time-point. Correlation coefficient and *P*-values are reported for significant correlations (Spearman *P*-value < 0.01). **B** Characterization of CBO_Turquoise, CBO_Black, CBO_Blue, and CBO_Brown gene modules. For each module, the ribbon plot visualizes the behavior of the module eigengene throughout differentiation time-points; each dot represents a data point, while the line connects time points of the same replicate (line and differentiation batch). Network reconstruction for the top-75 genes of each module, selected according to the intramodular connectivity value. Degree, closeness, betweenness, and eigenvector are represented by node label transparency, node color darkness, node border width, and node size/node label font size, respectively. Node shape represents gene biotype, with protein-coding genes as circles. **C** Bubble plot depicting the results of GO enrichment analysis for the four modules: *P*-values and enrichment scores are reported (details are reported in Supplementary Table 3).

To identify transcriptional patterns in CBO differentiation, we applied WGCNA and identified 14 co-expression modules (Supplementary Fig. 6A–C, Supplementary File 2). Correlation with differentiation stage uncovered as strongly correlated modules the CBO_Turquoise and CBO_Black (positive correlation) and CBO_Brown and CBO_Blue (negative correlation) (Fig. 3A). We analyzed the behavior of CBO_Turquoise and CBO_Black ME in the 8 samples across time-points (Fig. 3B) detecting a steady increase over time. While CBO_Turquoise modulation was reproducible across replicates, the CBO_Black was more variable, pointing to a small set of genes (180 genes against the 3279 of the CBO_Turquoise) with a less robust behavior. The variability of this subset of genes was observed mainly in 2 samples, which, however, showed patterns in line with the other samples for other modules.

GO enrichment analysis for CBO_Turquoise and CBO_Black retrieved terms related to neuronal fate commitment and maturation. Network reconstruction confirmed CBO_Turquoise hub genes as related to neurotransmission and synaptic function (e.g. NRXN2, AGAP2, SLC4AE, GABBR1, and GRM5), while the CBO_Black network comprised several transcription factors related to excitatory neuron identity (NEUROD6, SLA, BCL11B, RORB) (Fig. 3B, C). CBO_Brown and CBO_Blue ME were instead decreasing along differentiation; the first was associated to DNA replication and cell cycle, while the second to more general functions such as transcriptional and translational regulation (Fig. 3B, C). WGCNA also detected modules characterized by non-monotonic trends through differentiation. Among them, CBO_Green and CBO_Red showed levels dropping from Day25 to Day50 and increasing again at late stages. Both modules were enriched in genes related to cell adhesion and ECM organization (Supplementary Fig. 6D, E).

In summary, we time-resolved the transcriptional evolution of CBO by identifying co-expression patterns and transcriptional hubs driving their differentiation. We generated a knowledge base that classifies genes in distinct modules of behavior along organoid differentiation and links them to specific functional domains. We found overall consistency among lines from independent individuals and replicates. Main modules, their behavior, and relevance of every gene composing them are available in Supplementary File 2.

### Benchmarking of brain organoids against prenatal human corticogenesis reveals heterochronicity of differentiation across protocols

**Fig. 4:**
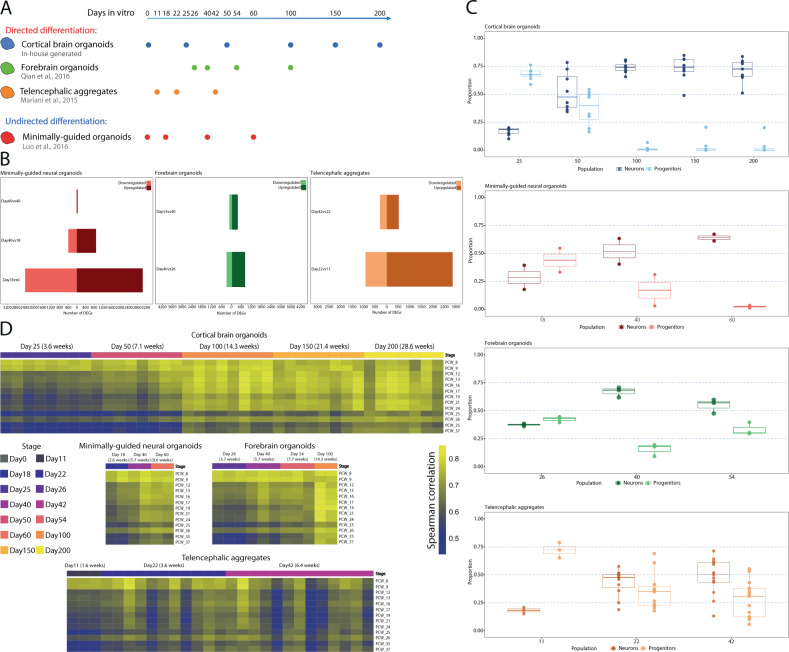
Brain organoids differentially recapitulated the timing of corticogenesis. **A** Schematic representation of in-house and external BO datasets and relative differentiation time points (CBO cortical brain organoids, FO forebrain organoids, TA telencephalic aggregates, MGO minimally-guided neural organoids). **B** Stage-wise differential expression analysis in MGO, FO, and TA. Barplots reporting the number of DEGs (FDR < 0.05, log2FC > 1 as absolute value), shown as upregulated and downregulated genes for each comparison. **C** Boxplots reporting the fraction of Progenitors and Neurons estimated by bulk deconvolution in each organoid dataset. **D** Correlation analysis of organoid datasets against the BS fetal cortex. Heatmaps visualize the correlation coefficient (Spearman correlation) for each organoid data point and each BS PCW.

To compare CBO against other protocols and evaluate them versus the fetal cortex, we selected three external BO datasets for which RNAseq was publicly available. These datasets of archetypal BO protocols, different for degree of guidance and culture conditions, included samples at comparable time-points (from 0 to 100 days). The selected BO datasets were: (i) minimally-guided neural organoids (MGO) [14]; (ii) forebrain organoids (FO) [20]; (iii) telencephalic aggregates (TA) [21], including a first step of 2D differentiation (Fig. 4A and Supplementary Fig. 7).

Stage-wise DEA revealed also for MGO, FO, and TA a decrease in the number of DEGs as differentiation progresses (Fig. 4B). However, the slow-evolving phase, which started between day 100–150 in CBO (Fig. 2C), was anticipated for all other protocols (day 40–60) (Fig. 4B). We then compared the DEGs identified for each stage transition in each external BO dataset to those retrieved for CBO (Supplementary Fig. 8A–C). The results highlighted a similarity in the transcriptional evolution of the models, with a high degree of overlap especially for MGO. In line with DEA, we found a more compressed time frame in the evolution of the other models compared to CBO, given the time points for which the overlap was significant.

Bulk deconvolution analysis focusing on two broad cell populations (early progenitors and neurons) showed progenitors as predominant in all models at early stages (Fig. 4C). In CBO, proportions of the two populations were comparable at day 50, with neurons prevalent from day 100 onwards. MGO, FO, and TA showed a similar but accelerated dynamic compared to CBO, again indicating a more rapid transcriptional maturation.

We then tested the transcriptional similarity of each model towards BS fetal cortex (Fig. 4D). Whole-transcriptome correlation showed for CBO a gradual increase of similarity towards mid and late PCW over time. We observed a more time-compressed evolution among MGO and FO, which already by day 60 showed an extent of similarity with late PCW that CBO reached only by day 100. For CBO and TA, the dataset encompassed organoids generated from different control individuals. CBO demonstrated robust reproducibility across genetic backgrounds and batches of differentiation, while TA were less tolerant to interindividual variability. These observations were further confirmed by using CBO as reference (Supplementary Fig. 8D).

Overall, several lines of evidence pointed towards heterochronicity across BO models in recapitulating the transcriptional modulations of corticogenesis. Although other experiments including more BO models and the same genetic backgrounds for all differentiation methods are needed to fully characterize this phenomenon, this is the first report of such differences in timings of differentiation of BO, which is an essential variable to control for pinpointing specific developmental transitions and disease phenotypes [42].

### Gene signatures specific for brain cell subpopulations and functions unveil dynamics of brain organoid differentiation

**Fig. 5:**
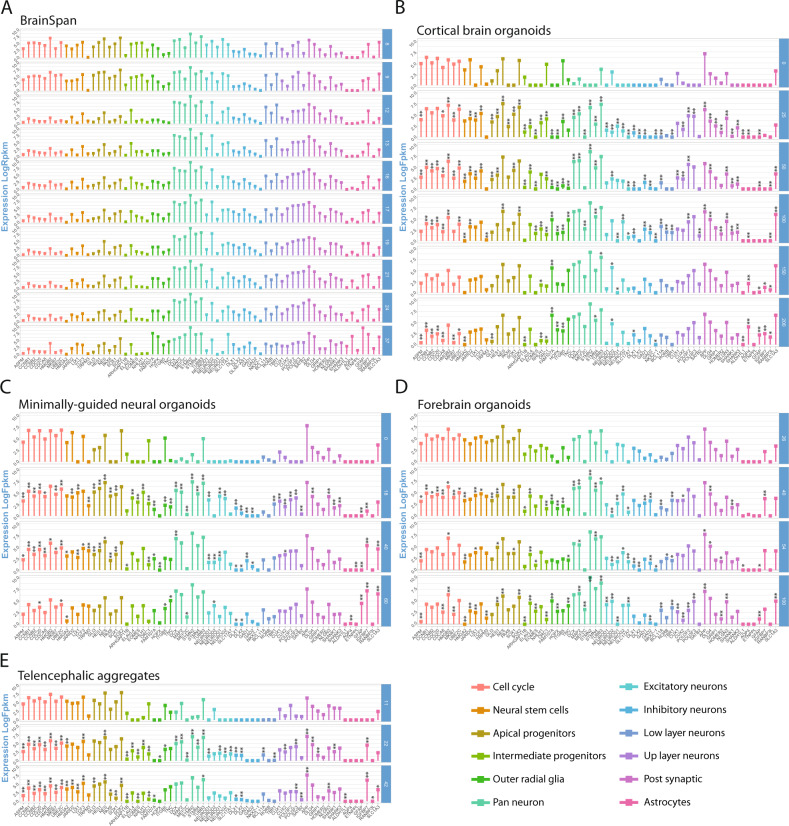
Gene expression levels of signatures related to cell population identity and function in BO. Expression levels (Log2Fpkm or Log2Rpm) in BS (**A**), CBO (**B**), MGO (**C**), FO (**D**), and TA (**E**) along time-points are reported in each dataset as the mean value across replicates. Each bar color corresponds to a specific signature, as reported in the plot legend. For brain organoids (**B**–**E**), asterisks identify genes that result significantly modulated in stage-wise differential expression analyses (comparison of each differentiation stage with the previous) reported in Fig. 2 for CBO and Fig. 4 for the other protocols. **: FDR < 0.05; *: Conventional *P*-value < 0.05.

We then examined how specific hallmarks of brain cell subpopulations and functions evolve during BO differentiation. To this end, we compiled a catalog of genes by manually curating new signatures from literature and by using signatures proposed in published studies [10, 15]. We observed their expression dynamics across BO and the developing cortex. Expression levels of signature genes are reported in Fig. 5 and Supplementary Figs. 9 and 10.

Fetal cortex showed drastic reduction of cell cycle, neural stem cell, apical progenitor, and intermediate progenitor gene levels between PCW 9-12, in line with WGCNA results. We found in BO models a less abrupt reduction of cell cycle and apical progenitor markers. The intermediate progenitor signature was found to peak at different time-points across models.

Excitatory neuron markers were particularly expressed at early PCW in the fetal cortex, a trend mirrored by CBO; MGO, FO, and TA showed a more persistent expression throughout differentiation.

oRG genes were well expressed in BS at PCW 8–9, decreased from PCW 12 and increased again at later stages, when astrocyte markers also appeared. CBO showed constant upregulation of the whole oRG signature starting from day 100, while for FO this was observed already at day 40; MGO and TA showed a partial expression at the available time-points.

Considering that the examined BO protocols were based on different degrees of guidance and culturing conditions, we then looked at brain area-specific genes, markers of off-target tissues, markers of neurotransmission, and stress-related genes (detailed in Supplementary Fig. 9, Supplementary Fig. 10, Supplementary Table 2). As markers of metabolic stress, we looked at the expression of glycolytic and ER-stress genes [15]. We found fluctuations of expression of those genes throughout cortical development as well as in BO, however, without indications of a sustained and strong increase at advanced time-points. In conclusion, by interrogating prenatal cortex and different BO datasets with literature-curated gene signatures, we highlighted biological domains for which each of these datasets manifested similarities to human fetal cortex development, as well as temporal peculiarities in modulating specific neural population markers.

### Brain organoids differentially capture distinct transcriptional patterns of fetal corticogenesis

The knowledge base of gene co-expression modules we reconstructed for corticogenesis and CBO differentiation allowed us to directly compare modules identified in cortex/CBO and follow their behavior in the other BO models. First, we measured the overlap between the two sets of WGCNA modules (Supplementary Fig. 11). Fetal BS_Turquoise, BS_Pink, BS_Midnight-blue, and BS_Grey60 genes, steadily increasing their expression along cortigogenesis, significantly overlapped with CBO_Turquoise, which showed the same trend. Similarly, BS_Yellow and BS_Black, functionally associated to cell cycle, showed steady decrease over time and overlapped with CBO_Brown, which had similar behavior and functional characterization (Supplementary Fig. 11).

We then sought to evaluate the relevance of the identified gene modules in the molecular circuitries that are affected in neurodevelopmental disorders and psychiatric conditions (Supplementary Fig. 12). We performed an overlap analysis to quantify the enrichment of genes from SFARI and Development Disorder Genotype - Phenotype Database (DD2P); moreover, we retrieved from the GWAS Catalog risk genes for four psychiatric disorders (Attention Deficit Hyperactive Disorder, Autism Spectrum Disorder, Schizophrenia, Unipolar Depression) and two non-psychiatric conditions (Diabetes Mellitus, Inflammatory Bowel Disease).

**Fig. 6:**
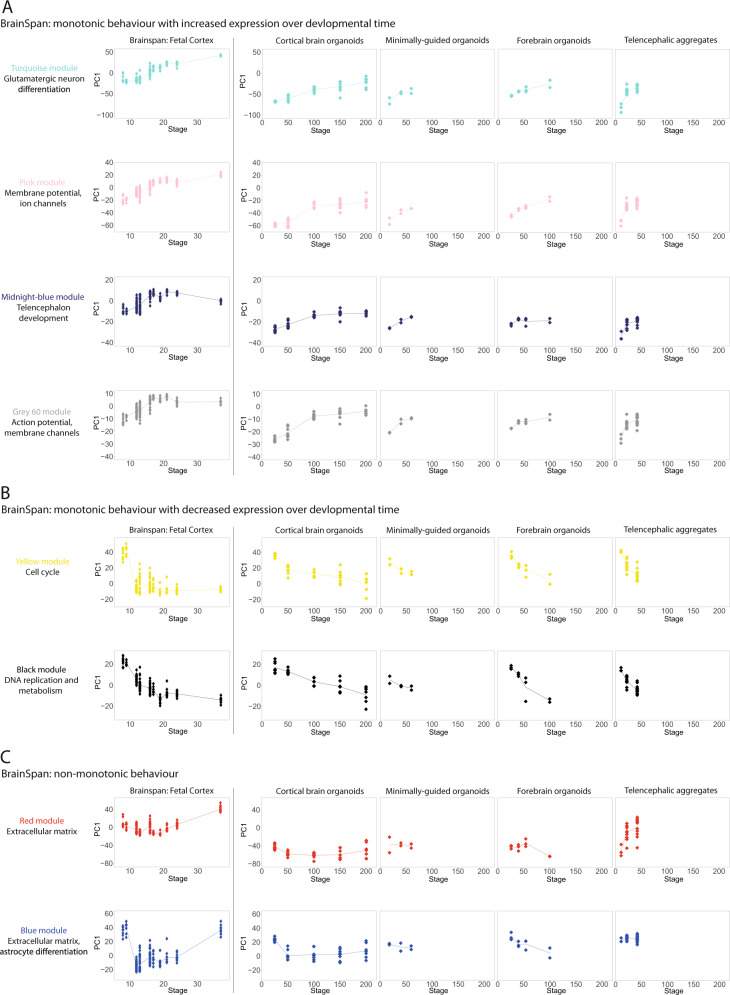
Behavior of the module eigengene for BrainSpan gene co-expression patterns in BO. **A** Visualization of BS_Turquoise, BS_Pink, BS_Midnightblue, and BS_Grey60 in prenatal fetal cortex as well as in the BO datasets. PCA was performed to calculate the module eigengene on the BrainSpan and then applied to each BO dataset. Each dot represents a data point, while the line connects the median value for each post-conceptional week (BS) or differentiation day (BO). The same analysis and visualization was applied to BS modules with a decreased expression along development (**B**) or with a non-monotonic behavior (**C**).

For the fetal cortex, we found a significant overlap found for the modules identified as related to neurogenesis and neural maturation (e.g. BS_Grey60, BS_Midnightblue, BS_Turquoise) and genes genetically associated to ASD from the SFARI repository, as well as risk genes for several psychiatric disorders (Supplementary Fig. 12A, C). Similarly, we identified the enrichment of genes associated to psychiatric conditions specifically in CBO gene modules associated to neuron commitment and maturation (CBO_Turquoise, CBO_Black, Supplementary Fig. 12B, D). We then analyzed the behavior of the same BS modules in each organoid dataset by following their ME across differentiation, revealing a close resemblance to fetal cortex trends (Fig. 6A, B). In a complementary approach, we applied the same analysis on the most relevant modules detected in CBO. The behavior of CBO_Turquoise and CBO_Brown in fetal cortex and other BO protocols was consistent with the one described in CBO. CBO_Black genes induction was variable across models and replicates (Supplementary Fig. 13A). CBO_Blue genes behaved similarly in all BO, and showed instead a non-monotonic behavior in BS, with peaks of expression at very early and very late PCW (Supplementary Fig. 13B).

Finally, we analyzed how BO recapitulated patterns of non-monotonic expression through time. BS_Red, BS_Blue, and CBO_Red, CBO_Green significantly overlapped between the two networks. The visualization of BS_Red and BS_Blue trends in BO revealed differences across protocols. CBO mostly displayed the same U-shape found in BS, although with a milder drop at intermediate stages. Conversely, MGO showed very little variation, FO showed a drop at day 100, and TA showed an opposite trend for the red module and no change for the blue one (Fig. 6C). Likewise, CBO and BS behaved similarly for the set of CBO_Red and CBO_Green genes, while these modules showed no, little or opposite variation in MGO, FO, and TA (Supplementary Fig. 13C). Among the functions of this set of modules, ECM and cell adhesion were prevalent.

Together, these results suggested that monotonic gene expression dynamics related to neuron specification and progenitor proliferation are shared between fetal cortex and CBO and are largely recapitulated by the analyzed BO paradigms. Conversely, trends of non-monotonic expression are largely recapitulated in CBO and less robustly in other BO systems at the examined time-points. The overlap between co-expression patterns of development/differentiation with genes related to neurodevelopmental and psychiatric disorders further substantiated the ability of our WGCNA approach to pinpoint gene modules that are meaningfully related from a functional and pathogenic point of view. This confirms the relevance of the identified networks for exploring key genes for the physiology and pathology of corticogenesis. Importantly, this analysis also confirmed the preservation of transcriptional programs relevant for disease modeling in CBO.

## Discussion

Despite the complexity of neuropsychiatric conditions, in terms of both phenotypic and time course heterogeneity, it is now well established that BO can allow the discovery of relevant developmental endophenotypes that are starting to illuminate disease pathogenesis with first inroads also in terms of patients’ stratification and drug discovery [8, 43]. The ambition is thus rapidly progressing from the initial focus on monogenic syndromes of high penetrance to the more high-throughput and challenging interrogation of polygenic loadings for mental health vulnerability, including in terms of the developmental antecedents of its later onset manifestations. Yet, the more central neurodevelopment becomes to our understanding of mental health, the more we need to gain a full understanding of its experimental recapitulation in vitro, so as to define clear benchmarks across systems and thereby empirically guide the most appropriate disease modeling designs. Towards this goal, here we first defined co-expression patterns characterizing human corticogenesis in vivo and in vitro and then organized them in interactive tables where gene relevance can be visualized, together with the overall behavior of these patterns along fetal cortex development and CBO differentiation (Supplementary File 1 and 2). The generation of this knowledge base allowed us to perform cross-comparisons between BS, CBO, and other BO paradigms, quantifying the preservation of co-expression patterns of corticogenesis in these models. To our knowledge, this represents the first resource of such kind. Similar analyses were performed on BS and BO [12, 14], however, without focus on prenatal cortex, accessibility to gene modules composition, or prioritization of transcriptional hubs (both known and novel). Thus, this knowledge base templates an open framework for the benchmarking of modeling studies and serves as a platform to interrogate when choosing the experimental system that best suits specific modeling needs or research questions. Several studies provided insights on the variability of BO, with considerable efforts of standardization. Our results demonstrated CBO reproducibility in recapitulating the transcriptional landscape of prenatal corticogenesis, in line with other reports [12, 44], with a developmentally relevant timing.

We observed that TA were more variable compared to CBO in terms of cell composition and global transcriptome when considering hiPSCs from different individuals. The same evaluation could not be carried out for MGO and FO due to the availability of a single genetic background.

Several analytical approaches (DEA, whole-transcriptome correlation with fetal cortex, bulk deconvolution) revealed a fast-evolving phase followed by a slow-evolving one during CBO differentiation. Among genes showing modulation also in the slow-evolving phase, we found upregulation of GABAergic neuron markers (such as CALB2, SCGN, SLC6A11), in line with previous studies [17, 38, 39, 45]. Among the functional categories emerging when analyzing the slow-evolving phase, we observed an upregulation of ECM-related genes. We also observed upregulation of genes related to mesoderm, however, the majority of them is expressed also in astrocytes/reactive astrocytes [34, 46, 47]. The developmental timing of astrocytes appearance in CBO (day 100–150—week 14.3–21.4) aligned with the human prenatal cortex. The interplay between neuronal and astrocytic cells is increasingly recognized in neuropsychiatric disorders [48–50], therefore the characterization of their differentiation in BO compared to fetal cortex is important to guide disease modeling.

In the limited time-frame available for external BO datasets, we confirmed their biphasic evolution. However, we observed heterochronicity among protocols. We compared similar in vitro ages for all protocols analyzed, with the CBO dataset including also very late stages, and observed that MGO, FO and TA reached the slow-evolving phase earlier than CBO, with a greater proportion of neurons at earlier time-points. In addition, MGO and FO globally correlated earlier with late stages of corticogenesis and CBO differentiation.

A possible explanation for these findings could be related to the maintenance of a more immature stage for longer time due to the prolonged use of EGF/bFGF during CBO differentiation, which, however, ended up matching more faithfully the in vivo counterpart. It is also known that iPSCs can have different propension to neuronal induction depending on the line itself and on culture conditions [51, 52]. However, both CBO and TA were generated from multiple hiPSCs lines reprogrammed from different individuals, thereby making improbable that differences across cell lines represent the main source of the observed heterochronicity among protocols. To our knowledge, this is the first description of such temporal dynamics in BO. Other reports comparing different paradigms focused on more heterogeneous time-points across protocols and other aspects of recapitulation [15–17]. Temporal dynamics recapitulation has remarkable relevance for disease-modeling, given the emergent convergence of molecular phenotypes of delay or acceleration in neuronal differentiation characterizing different clinical conditions [4, 53], and the eventuality of masking disease-relevant phenotypes if they are not correctly preserved [42]. Moreover, the tight link between neurodevelopment and risk/resilience of neuropsychiatric disorders positions the in vitro recapitulation of developmental timing at the core of the choice between BO systems, also with a view to balance the preservation of accurate developmental timing vis a vis the accelerated yield of more mature cell types involved in later onset neuropsychiatric disorders. Although further studies are needed to confirm and further dissect timing differences in BO, including multiple differentiation protocols from the same hiPSCs lines at the same time points, our analyses started to elucidate these dynamics, suggesting that CBO differentiation aligns with the in vivo temporality of corticogenesis.

Cellular stress in BO was previously reported by scRNAseq with the upregulation of glycolytic and endoplasmic reticulum stress signatures in several paradigms compared to primary samples [15, 16]. However, we did not observe a sustained increase of stress-related signatures along BO differentiation, corroborating the hypothesis of a homeostatic metabolic state in organoids rather than a deleterious stress increase over-time, in line with recent reports [11, 12].

Cross-visualization of monotonic BS co-expression patterns in CBO and vice versa revealed concordance in their behavior, a finding observed also for the other BO datasets. Overlap analysis with knowledge bases of genes associated to neurodevelopmental and psychiatric disorders indicated their selective enrichment in gene modules functionally associated to neuron fate commitment and maturation. Importantly, these modules were conserved from fetal brain to brain organoids, a relevant feature for disease modeling.

Modules with more complex temporal patterns were better recapitulated in CBO than in other protocols. Functional characterization of these modules pointed towards astrogenesis and ECM as predominant terms.

We also processed primary fetal tissues in-house, allowing direct comparisons against CBO because of the identical processing of the two datasets. The visualization of cortex-specific modulated genes in CBO highlighted again ECM. ECM genes have dynamic expression during corticogenesis, and have a role in regulating cortical folding, neuronal progenitor proliferation, and neuronal migration [54–56]. They have high expression in germinal zones and maturing/mature astrocytes [54, 57] and are important for the evolution of the human brain, with progenitor cells expressing ECM components at higher levels than in mice [58]. ECM also plays critical roles in synaptic plasticity and electrophysiological properties [59, 60], and its alteration has been linked to neuropsychiatric disorders [61, 62]. Our exposure of the differential regulation of ECM genes in BO compared to fetal cortex provides a relevant insight to consider when interpreting neuropsychiatric disease modeling datasets.

As already discussed, one limitation of this study, inherently due to the original design of the respective studies, is that the MGO and FO datasets derive from a single hiPSCs line. We therefore cannot exclude that the results we observed could be partially influenced also by the genetic background of the examined line for those BO models. Further studies are warranted to confirm the observations across different genetic backgrounds, building on the approaches we previously established for guiding optimal disease modeling designs by benchmarking transcriptional signal to noise detection vis a vis human genetic diversity [63]. Moreover, by inception this study did not aim to cover the complete landscape of BO protocols that have been developed so far. We focused our analyses on four selected protocols because of the range of epistemic objectives and attending culture conditions that they represent. While this could prima facie restrict the ordering reach of our work, we note that many if not most protocols currently used are characterized by minor adjustments and optimizations derived from these four archetypal protocols which, especially as far as CBO and MGO are concerned, represent the main forerunners and references of the field. Finally, the framework we have established in this work is easy to translate to other BO systems in follow-up studies and can thus be flexibly repurposed and harnessed, as the field progresses, as an analytical companion to the standardizing nomenclature effort we recently spearheaded [64].

Taken together, our results contribute to the definition of transcriptional footprints and dynamics specific to prenatal corticogenesis, representing a collection of prioritized known and novel hubs categorized in well-defined functional domains and made available as interactive tables (Supplementary File 1 and 2). Our approach describes the extent of in vivo/in vitro alignment of developmentally relevant processes and temporality, highlighting commonalities and differences across BO paradigms and providing a resource to be harnessed when modeling physiological or pathological human corticogenesis.

## Methods

### hiPSCs reprogramming and maintenance

Skin fibroblasts from four healthy individuals were reprogrammed using non-integrating self-replicating mRNAs as previously described [65] (Stemgent, 00-0071) (CTL1, CTL3, and CTL4) or Sendai virus (CTL2) (CytoTune-iPS 2.0 Sendai Reprogramming Kit; Thermo Fisher Scientific, A16517). Fibroblasts of CTL1 (phenotypically normal male without intellectual disability or other physician-diagnosed neuropsychiatric diagnosis) were received from BC Children’s Hospital, Vancouver. CTL2 (male) hiPSC line was received from the Wellcome Trust Sanger Institute. CTL3 (ShiPS-MIFF1 [66], male) hiPSC line was received from the university of Sheffield. Fibroblasts of CTL04 (female) were received from the GGDBbank, member of the Telethon Network of Genetic Biobanks (project no. GTB12001), funded by Telethon Italy, and of the EuroBiobank network. hiPSC were cultured in TeSR-E8 medium (Stemcell technologies, 05990), with daily media change, at 37 °C, 5% CO_2_, and 3% O_2_ in standard incubators. hiPSC were grown on matrigel-coated dishes (Corning, 354248) and passaged using ReLeSR^TM^ (Stemcell technologies, 05872). Cell lines were routinely profiled through STR analysis to determine authenticity and to mycoplasma test. All lines were negative to mycoplasma. All subject signed an informed consent and the use of hiPSCs was approved by the ethical committee of the University of Milan.

### CBO differentiation

CBO were generated using an adaptation of the previously described protocol published by Pasca et al. in 2015 [19], introducing orbital shaking on day 12 of differentiation as described in [18]. Briefly, hiPSC were grown on feeders for 3–4 days in a medium composed of 80% DMEM/F12 medium (1:1), 20% Knockout serum (Thermo Fisher Scientific, 10828028), 1% Non-essential amino acids (NEAA, Lonza BE13-14E), 0.1 mM cell culture grade 2-mercaptoethanol solution (Thermo Fisher Scientific, 31350010), 2 mM L-Glutamine (Thermo Fisher Scientific, 25030081), P/S 100 U/mL, and FGF2 at a final concentration of 20 ng/mL (Thermo Fisher Scientific, PHG0021). Daily media change was performed. Embryoid bodies (EB) were generated by detaching hiPSC with dispase for 40 min and plating on ultra-low attachment 10 cm plates (Corning, 3262) in the first differentiating medium composed of 80% DMEM/F12 medium (1:1), 20% Knockout serum, 1% NEAA, 0.1 mM cell culture grade 2-mercaptoethanol solution, 2 mM L-Glutamine, P/S, 100 U/mL, 7,5 μM Dorsomorphin (MedChem express, HY-13418A), 10 μM TGFβ inhibitor SB431542 (MedChem express, HY-10431), and ROCK inhibitor 5 μM. EB were grown in normal oxygen incubators. EB were left undisturbed for 1 day and at 48 h media change was performed with differentiation medium 1 without ROCK inhibitor. Dorsomorphin and TGFβ inhibitor are used to perform Dual-SMAD inhibition, pushing neuroectoderm specification. Dual-SMAD inhibition was performed for a total of 5 days, with daily media change. On day 6 the second differentiation medium was added until day 25 with daily media change for the first 12 days, and then every other day. The second differentiation medium was composed of neurobasal medium (Thermo Fisher Scientific, 12348017) supplemented with 1X B-27 supplement without vitamin A (Thermo Fisher Scientific 12587001), 2 mM L-Glutamine, P/S, 100 U/mL, 20 ng/mL FGF2, and 20 ng/mL EGF (Thermo Fisher Scientific, PHG0313). Human FGF2 and EGF were used to amplify the pool of neural progenitors. On day 12, CBO were moved to ultra-low attachment 10 cm dishes and grown on shakers to enhance oxygen and nutrient supply. On day 26, FGF2 and EGF were replaced with 20 ng/mL brain-derived neurotrophic factor (BDNF, Peprotech 450-02) and 20 ng/mL neurotrophin-3 (NT3, Peprotech 450-03) to promote differentiation of neural progenitors towards the glutamatergic fate. From day 43 onwards, BDNF and NT3 were removed and from day 50 the medium was supplemented with Amphotericinβ to prevent mold formation. Before processing for transcriptomics, organoids were visually inspected and only organoids with round shape and with comparable size among lines were selected. Each line was differentiated and profiled at the following time points: iPSC stage (Day0), Day25, Day50, Day100, Day150, and Day200, in two different rounds of differentiation. Both differentiation rounds were profiled for each line, except for Day 200 for one of the lines, for which only one replicate was available. The cohort of sample is therefore composed by a total of 43 samples (39 organoids plus 4 iPSCs).

### Immunostainings of CBO

CBO were harvested on day 25, 50, 100, and fixed 2 h in paraformaldehyde 4% (ChemCruz, sc-281692) on orbital shaking. Then, sucrose 30% was added for 12–16 h after a wash in PBS 1X. CBO were then embedded in cryostat embedding medium (Bio Optica 05-9801). Cryosection was obtained with a standard protocol using Leica CM 1900 instrument with 10 μm thickness. Sodium Citrate Buffer (10 mM Sodium Citrate, 0.05% Tween, in ddH_2_O, pH 6.0) was used for antigen retrieval. Slides were immersed in Sodium citrate buffer in the water-bath at 95 °C for 45 min and then left at room temperature for 45 min. Slides were then treated with 0,1 M Glycine pH 7.4 for 10 min to reduce autofluorescence. Subsequently, blocking solution was added (5% serum, 1% triton X-100 in PBS 1X) and the slides were incubated 30 min at room temperature. Primary antibodies in antibody buffer (2% South America serum, Euroclone ECS0182L, in PBS 1X) were added and slides were incubated ON at 4 °C. The day after, 5 consecutive washes in PBS were performed and then the slides were incubated for 1 h at room temperature with the secondary antibodies in antibody buffer. DAPI (Sigma, D9542) was added for 5 min at room temperature after 3 washes in PBS 1X. Slides were washed with PBS 1X and then water, dried and mounted using VectaMount mounting media (Vector Laboratories, H-5501). The following primary antibodies were used: BCL11B, 1 in 200, Rat, Abcam, ab18465; GFAP, 1 in 50, Rabbit, Sigma, G9269; KI67, 1 in 500, Rabbit, Abcam, ab15580; PAX6, 1 in 250, Rabbit, Biolegend, 561664; SOX2, 1 in 50, Goat, R&D system, AF2018; SATB2, 1 in 100, Mouse, Abcam, ab51502; TBR2, 1 in 200; Rabbit, Abcam, ab23345. All images were acquired on a widefield microscope (Leica DMI6 B), equipped with an Andor Zyla (VSC-04470 sCMOS), using 20X/0.75 dry objective.

### Culture conditions for fetal cortical cell

Human fetal neural stem cell cultures were derived and maintained as previously described [5, 67]. They were derived from the cortex of WGA 11 and 19, male embryos. They were cultured in the following medium DMEM/F12 medium (1:1), P/S (100 U/mL), 0.1 mM cell culture grade 2-βmercaptoethanol solution, 1% NEAA, 0.5% N_2_ supplement, 1X B27 Supplement 100X (Thermo Fisher Scientific, 17504-044), 0.012% Bovine Albumin Fraction V (Thermo Fisher Scientific, 15260-037), 1.5 g/L glucose (Sigma-Aldrich, G8644). Washes for this type of cells were performed with a medium composed of DMEM/F12 medium (1:1), P/S (100 U/mL) and 0.015% Bovine Albumin Fraction V.

### Download of external datasets

BrainSpan dataset: pre-processed Rpkm values for the BrainSpan Atlas were downloaded from here: http://www.brainspan.org/static/download.html. Dataset organisation was described in the following white paper: https://help.brain-map.org/display/devhumanbrain/Documentation, Developmental Transcriptome.

MGO, FO and TA datasets: bulk RNA sequencing data were downloaded from Gene Expression Omnibus using the relative article accession numbers (GSE82022, GSE80073, GSE61476, respectively). These protocols were selected because they represent a wide range of guidance and culture conditions. Moreover, they are references from which several derivations have been developed by several labs, with many of them being characterized by minor adjustments of these archetypal protocols which, especially for CBO and MGO, represent the main forerunners and references of the field.

### RNA extraction and library preparation for RNA-seq

Total RNA was extracted from snap-frozen pellets of CBO at day 25, 50, 100, 150, 200, fetal cortical progenitors and fetal brain tissues using the Rneasy Mini Kit (Qiagen, 74104). Purified RNA was quantified using a NanoDrop spectrophotometer and RNA quality was checked with an Agilent 2100 Bioanalyzer using the RNA nano kit (Agilent, 5067-1512). Library preparation for RNA sequencing was performed according to TruSeq Total RNA sample preparation protocol (Illumina, RS-122-2202), starting from 250 ng to 1 μg of total RNA. cDNA library quality was assessed with an Agilent 2100 Bioanalyzer, using the high-sensitivity DNA kit (Agilent 5067-4626). Libraries were sequenced with the Illumina Novaseq machine at a read length of 50 bp paired-end and a coverage of 35 million of reads per sample.

### RNAseq quantification for CBO, in-house fetal dataset, external brain organoids datasets

RNAseq FASTQ data were quantified at the gene level using Salmon (version 0.8.2 [68]). GRCh38 Genecode 27 was used as reference for quantification and annotation.

### BrainSpan correlation analysis across developmental stages

The analysis was applied on BS specimens from prenatal and post-natal cortex. After selecting protein-coding genes, the mean expression was calculated for each stage (taking into account all the samples and sub-areas). Spearman correlation across samples was calculated and the correlation coefficient was visualized by heatmaps using pheatmap R package (https://CRAN.R-project.org/package=pheatmap).

### Principal component analysis

BrainSpan: after selecting only samples from prenatal cortex, a filtering to discard not-expressed or low-expressed genes was applied by keeping genes with expression of at least 1 Rpkm in at least 1/4 of the samples (16,824 genes selected).

Internal fetal dataset and CBO: a total of 66 samples considering CBO and internal fetal samples was used to perform PCA. Gene filtering using a threshold of 2 counts per million reads (cpm) in at least 2 samples was used, resulting in 17,759 analyzed. CBO dataset: a total of 39 samples considering CBO dataset (from day 25 to day 200). Gene filtering using a threshold of 2 cpm in at least 4 samples was used, resulting 16,901 genes used for the analysis.

For all datasets, PCA was computed using R prcomp function. For both BS and CBO PCA, gene loadings for PC1 and PC2 were retrieved from this analysis and the top 35 ones with positive and negative scores were visualized as lollipop graphs. The top 300 genes with the highest positive loading and the top 300 with the highest negative loading were selected to perform GO for biological processes using the TopGO package [69], relying on Fisher test and Weight01 method. *P*-value < 0.01 and enrichment >1.75 were used as thresholds to select significantly enriched GO terms.

### WGCNA

#### BrainSpan

*Weighted Gene Co-expression network generation and module identification:* After selecting only samples from prenatal cortex, not-expressed or low-expressed genes were discarded by keeping genes with expression of at least 1 Rpkm in at least 1/4 of the samples (16,824 genes selected). Samples from post-conceptional weeks 25, 26, and 35 were identified as outliers by the sample clustering, and therefore excluded from downstream analyses. Starting from this set of 16,824 genes measured in 157 specimens, a gene selection strategy was applied by calculating for each gene the coefficient of variation (CV) across the experimental conditions after log-transformation. A 65-percentile threshold was then imposed, thus selecting the 35% of genes showing the highest values of CV (5889 genes). A signed gene co-expression network was generated relying on WGCNA R package (version 1.64.1 [70]). The correlation matrix was calculated by applying a biweight mid-correlation and then transformed into an adjacency matrix by raising it to the power of *β* = 18. Topological Overlap Measure was calculated from the adjacency matrix and the relative dissimilarity matrix was used as input for average-linkage hierarchical clustering and gene dendrogram generation. Network modules were detected as branches of the dendrogram by using the DynamicTree Cut algorithm (deepSplit = 1; minimum cluster size = 50; PAM stage TRUE; cutHeight 0.998 [71].

*Module-trait correlation:* As phenotypic trait for module-trait correlation, module eigengenes were related to each sample developmental stage (PCW), considered either as a continuous quantitative variable or dichotomized as a series of categorical variable (dummy variables).

*Module functional analysis:* Gene ontology enrichment analysis for the Biological Process domain was performed on the genes belonging to each module of interest, using the list of 5889 genes selected for network generation as custom reference set. Analysis was performed by TopGO as described above. *P*-value < 0.01 and enrichment >1.75 were used as thresholds to select significantly enriched GO terms.

*Sub-network visualization and analysis in Cytoscape:* Node and edge information for the selected modules were exported from the adjacency matrix, selecting for each module the top-75 according to the intramodular connectivity and imposing a threshold of 0.2 as minimum edge weight. Nodes and edges for each module were therefore imported in Cytoscape (version 3.8.2) for centrality analysis (CytoNCA [72]).

#### CBO

The same pipeline used to perform WGCNA on BS was applied also to the CBO dataset. The analysis was performed on a total of 39 samples with in vitro age spanning from day 25 to day 200.

We excluded from differential expression analysis genes with biotypes that were not of interest (e.g. ribosomal RNA, antisense, snoRNA), and focused prevalently on protein_coding and long intergenic non-coding genes). Filtering on gene expression was applied by keeping genes with an expression of at least 2 cpm in at least 7 samples (15,663 genes selected). Coefficient of variation was calculated on log-transformed data and was set to 0.5, resulting in a total of 7831 genes considered for the analysis. The soft threshold β was set to 15. The DynamicTree Cut algorithm parameters used for gene module identification were DeepSplit of 1; minClusterSize 30; PAM stage TRUE; cutHeight 0.999, for a total of 14 modules that were then characterized using the same approach described for BS.

For the generation of the bubble plot relative to CBO turquoise, black, blue and brown modules, REVIGO web tool was employed to summarize GO terms [73].

### Differential expression analyses (DEA)

CBO: DEAs were performed for CBO in a stage-wise approach comparing each differentiation stage with the previous time-point using edgeR 3.20.9. Genes with expression levels higher than 2 cpm in at least 4 samples were tested for differential expression (16,901 genes); after calculation of normalization factors, normalization and estimation of dispersion, differential expression was tested by glmFit approach. The information about line identity was used as a covariate in the statistical model. DEGs were selected imposing as thresholds FDR < 0.05 and absolute log2FC > 1. For each comparison, the number of DEGs, splitted in upregulated or downregulated, was represented by barplots. Functional enrichment analysis for the Biological Process domain of GO was performed by TopGO for every comparison dividing genes in up- and downregulated as setting the following parameters: ‘weight01’ as algorithm, ‘fisher’ as statistics and 15 as ‘nodeSize’ and 15 as ‘minTerms’.

*P*-value < 0.01 and enrichment >2 were used as thresholds to select significantly enriched GO terms.

Visualization of DEA results between the different sequential CBO comparisons was performed using scatter plots visualizing the log2FC of all tested genes; the color-code was set according to FDR values. Gene resulting differentially expressed in both sequential CBO comparisons (e.g. day 50vs25 against day 100vs50) were identified and divided in 4 quadrants. In this way, the behavior of genes in common between the two DEAs was analyzed, thus finding genes upregulated in both, downregulated in both or upregulated in one and downregulated in the other. The top 10 protein-coding genes in terms of absolute fold change for the four types of behaviors were labeled in the plot.

MGO, FO, and TA datasets: the same stage-wise DEA approach was applied for MGO, FO, and TA, analyzing each dataset independently. Genes with expression levels higher than 2 cpm in at least 2 samples were tested for differential expression with edgeR 3.20.9 (MGO: 15,339; FO: 16,585; TA: 16,522 genes). Line identity was used as a covariate for the TA dataset, while for MGO and FO only one line was available. DEGs were selected and visualized as described for CBO.

DEGs overlap: DEGs identified by stage-wise analysis in CBO were compared to the results of the same analysis on the other BO protocols. Overlap significance was tested by GeneOverlap R library (version 1.30.0, R version 4.1.1), considering the genes tested in CBO as universe. To retrieve transcriptional modulations robustly similar across protocols, we imposed stringent thresholds, selecting overlaps having *P*-value < 0.01 and odds ratio >3. Results were visualized by heamaps produced by the drawHeatmap function of the package. Only values with *P*-value < 0.01 and OR > 3 have been kept in the heatmap.

### Cortex-specific genes determination and visualization of their behavior in CBO

DEAs of the different fetal tissues from our internal cohort versus hiPSC were performed for all areas including at least two samples using edgeR 3.20.9. Genes with expression levels higher than 2 cpm in at least 2 samples were tested for differential expression (17,275 genes). DEGs with FDR < 0.05 and absolute log2FC > 3 were considered for further analysis. Cortex-specific DEGs were determined by subtracting to DEGs of the cortex vs hiPSC comparison DEGs found in the analysis between other tissues and hiPSC. Functional enrichment analysis for GO biological processes was performed by TopGO for cortex-specific genes dividing them in up- and downregulated. *P*-value < 0.01 and enrichment >2 were used as thresholds to select significantly enriched GO terms. The behavior of cortex-specific DEGs in the CBO dataset, including hiPSC, was visualized by heatmap using average-linkage hierarchical clustering for rows.

### Bulk deconvolution

Proportions of cell populations were estimated applying a deconvolution approach based on the SCDC algorithm [74] using bulk-RNASeq counts as input and a scRNAseq dataset of the developing human cortex [40] as reference. The single cell raw count expression matrix was filtered to discard low-quality cells, by keeping cells compliant with the following threshold: mitochondrial RNA content <5%; ribosomal protein RNA content <50%; detected genes >450 and <3000; UMI counts >750 and <10,000: after filtering, 14,610 genes measured in 27,527 cells were selected for downstream steps Starting from the clustering annotation performed in the original work, pericytes, microglia, endocytes and oligodendrocyte progenitors were not included, as we did not expect to find these cell type in CBO. The remaining cell populations were then grouped in the following 6 categories: ventricular Radial Glia (vRG); outer Radial Glia (oRG); Intermediate Progenitos (IP); cycling progenitors; interneurons: excitatory neurons. After deconvolution and for visualization purposes, the results were further grouped in two broader categories: progenitors (cycling progenitors + vRG) and neurons (excitatory neurons + inhibitory neurons). Cells with uncertain cell-type assignments were removed (SCDC qc threshold = 0.65). Cell proportions were retrieved using the SCDC_prop function and were visualized as boxplots showing the proportion of every cell type per stage.

Analysis was performed in R, version 3.6.1.

### Correlation of BO transcriptome versus BS or versus CBO

Transcriptome-wide correlation was calculated on Fpkm (Rpkm for BS) after selecting protein-coding genes and filtering out lowly-expressed ones (mean expression levels lower than 1 Rpkm in at least 75% of samples (BrainSpan) and 1 Fpkm (Brain Organoids)). Mean expression per gene per stage was calculated for all BS prenatal cortical samples. Correlation was computed in R using Spearman metrics and visualized as separated heatmaps for each comparison. The same approach was applied to perform correlation of external brain organoid datasets against CBO.

### Literature-curated gene signatures visualization in BS and BO

Expression values (in log2Fpkm or log2Rpkm) for the gene signatures of interest were retrieved and the mean expression was calculated for each stage of every dataset, then visualized by lollipop plots.

### Module overlap of BS and CBO WGCNA

Genes shared across the CBO and BrainSpan networks were selected for the analysis (2643); overlap across modules of interest was performed. Number of shared genes, odds ratio, and *P*-value across CBO and BrainSpan modules were calculated by the GeneOverlap R package. Results were visualized as dot plot were numbers (shared genes) were shown for odds ratio >1, dots were shown for those having also *P*-value < 0.05. Color-code was assigned according to OR, dot size varied according to *P*-value.

### Overlap of WGCNA gene modules and gene-disorder knowledge bases

The following gene-phenotype knowledge bases were considered for the overlap analysis: (I) Development Disorder Genotype–Phenotype Database (DD2P); (II) SFARI Genes; (III) the GWAS Catalog (EBI). SFARI and DD2P were downloaded respectively from https://gene-archive.sfari.org/tools/ and https://www.deciphergenomics.org/ddd/ddgenes. For DD2P, the overlap was tested with the complete database as well as on a subset related to the CNS, obtained by filtering the term ‘brain’ in the ‘Organ’ field.

GWAS catalog (https://www.ebi.ac.uk/gwas/) was interrogated to retrieve risk genes for 4 psychiatric disorders and two non-psychiatric conditions by searching for the indicated disease code: Attention Deficit Hyperactive disorder (EFO_0003888); Autism Spectrum Disorder (EFO_0003756); Schizophrenia (MONDO_0005090); Unipolar Depression (EFO_0003761); Diabete Mellitus (EFO_0000400); Inflammatory Bowel Disease (EFO_0003767).

Overlap was performed for both Brainspan and CBO WGCNA gene modules, selecting the ones identified as the most interesting from correlation analysis: CBO_black, CBO_blue, CBO_brown, CBO_green, CBO_red, CBO_turquoise modules for cortical organoids and BS_black, BS_blue, BS_grey60, BS_midnightblue, BS_pink, BS_red, BS_turquoise, BS_yellow for the fetal cortex. Overlap significance was tested by GeneOverlap R library (version 1.30.0, R version 4.1.1). Overlaps were considered significant with an odds ratio (OR) higher than 1 and *P*-value lower than 0.01. Results were visualized as dot plot with numbers (shared genes) shown for OR > 1, dots shown for those having also *P*-value < 0.01. Color-code was assigned according to OR, dot size varied according to *P*-value.

### Analysis of BS modules in BO and of CBO modules in BS and other brain BO

Module eigengene for each BrainSpan WGCNA module of interest was calculated in all organoid datasets as a prediction (R function predict) based on the module eigengene of BrainSpan itself. Likewise, module eigengene for each CBO WGCNA module of interest was calculated and predicted in BrainSpan and in external brain organoid datasets. Results were visualized as ribbon plots showing first principal component coefficients for each module along BrainSpan and organoid developmental time points.

### Statistical analyses

Unless otherwise specified, all bioinformatic analyses were performed using R version 3.4.4 except for the bulk deconvolution analysis performed using R version 3.6.1. The statistical details of all analysis can be found in the relative figure legend and text of the “Results” section.

## Supplementary information

Supplementary information file

Supplementary table 1

Supplementary table 2

Supplementary table 3

Supplementary file 1

Supplementary file 2

## Data Availability

Bulk RNAseq data generated in this study have been deposited in ArrayExpress and will be made available upon publication at the accession number E-MTAB-11239. No new algorithms were developed in this work.

## Code availability

Code snippets for data processing and characterization/visualization of gene modules included as supplementary files.
