# Supplementary material for: Benchmarking brain organoid recapitulation of fetal corticogenesis
Source: Transl Psychiatry. 2022 Dec 20;12:520. doi: 10.1038/s41398-022-02279-0 (PMC9767930; doi:10.1038/s41398-022-02279-0)
Supplement: Supplementary file 1 — Supplementary information file [file 41398_2022_2279_MOESM1_ESM.pdf]

Supplementary information

Benchmarking brain organoid recapitulation of fetal corticogenesis

Cristina Cheroni[1,2,3,#], Sebastiano Trattaro[1,2,3,#], Nicolò Caporale[1,2,3], Alejandro López-Tobón[1,2,3,4], Erika Tenderini[1,5], Sara Sebastiani[1,6], Flavia Troglio[1,2,3], Michele Gabriele[1,2,7], Raul Bardini Bressan[8], Steven M Pollard[8], William T Gibson[9,10], Giuseppe Testa[1,2,3,*]

[1] Department of Experimental Oncology, European Institute of Oncology IRCCS, Milan, Italy.
[2] Department of Oncology and Hemato-Oncology, University of Milan, Via Santa Sofia 9, 20122, Milan, Italy.
[3] Human Technopole, Viale Rita Levi-Montalcini 1, 20157, Milan, Italy.
[4] Current address: Herophilus, Inc., San Francisco, CA 94107, USA.
[5] Current address: Telethon Institute of Genetics and Medicine (TIGEM), Pozzuoli, Naples, Italy, Via Campi Flegrei, 34 - 80078.
[6] Current address: Department of Molecular Neurobiology, Donders Institute for Brain, Cognition and Behavior, Radboud University Nijmegen, The Netherlands.
[7] Current address: Department of Biological Engineering, Massachusetts Institute of Technology, Cambridge, MA 02139, USA; The Broad Institute of MIT and Harvard, Cambridge, MA 02139, USA; Koch Institute for Integrative Cancer Research, Cambridge, MA 02139, USA.
[8] Centre for Regenerative Medicine, Institute for Regeneration and Repair, and Edinburgh Cancer Research UK Centre, University of Edinburgh, Edinburgh, UK. EH16 4UU.
[9] BC Children's Hospital Research Institute, 950 West 28th Avenue, Vancouver, BC, Canada V5Z 4H4.
[10] Department of Medical Genetics, University of British Columbia, Vancouver, BC, Canada V6T 1Z4.
[#] These authors contributed equally.
*Correspondence: giuseppe.testa@fht.org; giuseppe.testa@unimi.it; giuseppe.testa@ieo.it.

Content:

- Supplementary figure and table legends
- Supplementary figures

Supplementary Figure 1

(**A-B**) Correlation analysis of BrainSpan prenatal and postnatal cortices. The heatmaps show the correlation coefficient calculated by Spearman correlation across stages, for prenatal (A) and postnatal (B) timepoints, respectively. (**C-D**) Top-35 genes according to positive and negative loading values for the first (C) and second (D) components from PCA analysis. Results of gene ontology enrichment analysis performed for the Biological Process domain on the top genes selected on the basis of their loading value are visualized in the bar plots.

Supplementary Figure 2

(**A**) Gene dendrogram generated on the Topological Overlap Dissimilarity matrix for BS cortex samples; each branch of the dendrogram corresponds to a module of highly interconnected genes, for a total of 17 modules. The row below the dendrogram depicts the module assignment, each identified by a different colour. (**B**) Sample dendrogram showing the clustering of Brainspan samples included in WGCNA analyses. The rows below the dendrogram depict post-conceptional week associated to each sample, either as continuous or categorical variable (first and other rows respectively). (**C**) Brainspan WGCNA diagnostic plots for the selection of beta parameter (set to 18) according to mean connectivity and scale independence values. (**D**) Ribbon plot, GO enrichment analysis and network reconstruction for BS_Midnightblue module.

Supplementary Figure 3

(**A**) Widefield images showing the expression of apical progenitor (PAX6) and neural stem cell (SOX2) markers in day 25 CBO of a representative control line. (**B**) Widefield images showing the expression of cell cycle (KI67), neural stem cell (SOX2), apical progenitor (PAX6), intermediate progenitor (EOMES) and low layer neuron (BCl11B) markers in day 50 CBO of representative control lines.

Supplementary Figure 4

(**A**) Widefield images showing the expression of apical progenitor (PAX6), neural stem cell (SOX2), low layer neuron (BCL11B), up layer neuron (SATB2) and astrocyte (GFAP) markers in day 100 CBO of representative control lines.

Supplementary Figure 5

(**A**) PCA on the CBO cohort, with differentiation stage shown as dot color. (B-C) Top-35 genes according to positive and negative loading values for the first (**B**) and second (**C**) components from PCA analysis and barplots showing functional enrichment results for the Biological Process domain on the top genes. (**D-E**) Functional analysis of up-regulated (D) and down-regulated (E) genes for Day150 vs Day100 and Day200 vs Day150 stage-wise differential expression analysis on CBO. (**F**) Scatterplots representing the relationship of the fold-change across successive time windows. Genes resulting differentially expressed (FDR < 5% and absolute log2FC > 1) in both the two examined comparisons are reported in yellow, while the ones specific for one of the two comparisons are in blue or purple. Gene symbols are reported for the top 10 protein-coding genes for each quadrant according to fold-change.

Supplementary Figure 6

(**A**) Gene dendrogram generated on the Topological Overlap Dissimilarity matrix for CBO samples; each branch of the dendrogram corresponds to a module of highly interconnected genes, for a total of 14 modules. The row below the dendrogram depicts the module assignment, each identified by a different colour. (**B**) Sample dendrogram showing the clustering of CBO samples. The rows below the dendrogram depict the differentiation day associated to each sample, either as continuous or categorical variable (first and other rows respectively). (**C**) CBO WGCNA diagnostic plots for the selection of beta parameter (set to 15) according to mean connectivity and scale independence values. (**D-E**) Ribbon chart, bubble plot and network reconstruction for the green (D) and red (E) modules.

Supplementary Figure 7

(**A**) Schematic representation of the differentiation steps of the four BO protocols analyzed. (**B-D**) PCA of the MGO (B), FO (C) and TA (D) datasets.

Supplementary Figure 8

Overlap between genes retrieved as differentially expressed by stage-wise differential expression analysis in cortical brain organoids (CBO) compared to external brain organoid dataset (Minimally Guided Organoids in panel **A**; Forebrain Organoids in panel **B**: Telencephalic Aggregates in panel **C**). P-value and Odds Ratio are reported for the overlap with P-value < 0.01 and Odds Ratio > 3. Cell color is assigned according to Odds Ratio values. (**D**) Heatmap depicting the results of the whole-transcriptome correlation analysis between CBO data and external BO protocols.

Supplementary Figure 9

Lollipops showing the expression levels of gene signatures related to brain areas, off-target tissues and cell stress in BS (**A**), CBO (**B**), MGO (**C**), FO (**D**) and TA (**E**). Expression levels (Log2Fpkm or Log2Rpm) along timepoints are reported in each dataset as the mean value across replicates. Each bar colour corresponds to a specific signature, as reported in the plot legend. For brain organoids (B-E), asterisks identify genes that are retrieved as significantly changed in stage-wise differential expression analyses (comparison of each differentiation stage with the previous) reported in Figure 2 for CBO and Figure 4 for the other protocols. **: FDR < 0.05; *: Conventional pvalue < 0.05.

Supplementary Figure 10

Lollipops showing the expression levels of gene signatures related to neurotransmission systems in BS (**A**), CBO (**B**), MGO (**C**), FO (**D**) and TA (**E**). Expression levels (Log2Fpkm or Log2Rpm) along time-points are reported in each dataset as the mean value across replicates. Each bar colour corresponds to a specific signature, as reported in the plot legend. For brain organoids (B-E), asterisks identify genes that are retrieved as significantly changed in stage-wise differential expression analyses (comparison of each differentiation stage with the previous) reported in Figure 2 for CBO and Figure 4 for the other protocols. **: FDR < 0.05; *: Conventional P-value < 0.05.

Supplementary Figure 11

Overlap between BrainSpan and Cortical Brain Organoid gene modules identified by WGCNA. Dot plot displaying the overlap between BS (y-axis) and CBO (x-axis) gene modules identified by WGCNA. Numbers represent shared genes and are shown for overlaps with odds ratio (OR) > 1, while dots are reported for those having also P-value < 0.05. Dot colour is assigned according to OR values and dot size according to P-value.

Supplementary Figure 12

Overlap between BrainSpan (A, C) or Cortical Brain Organoid (B, D) WGCNA relevant gene modules and gene-disorder knowledge bases. Panels A and B show the overlap with SFARI genes (SFARI) and Development Disorder Genotype - Phenotype Database (DD), either for all organs or selectively for brain (DD_B). Panels C-D represent the overlap with risk genes retrieved from GWAS catalogue for the following disorders: Attention Deficit Hyperactive Disorder (ADHD), Autism Spectrum Disorder (ASD), Diabetes Mellitus (DM), Inflammatory Bowel Disease (IBD), Schizophrenia (SCZ), Unipolar Depression (UD). In each dot plot WGNA gene sets are reported on the x-axis and disorder gene sets on the y-axis. Numbers represent shared genes and are shown for overlaps with odds ratio (OR) > 1, while dots are reported for those having also P-value < 0.01. Dot colour is assigned according to OR values and dot size according to P-value as shown by each legend.

Supplementary Figure 13

Behaviour of the module eigengene for Cortical Brain Organoids gene co-expression modules in fetal cortex and brain organoids datasets. (**A**) Visualization of CBO_Turquoise and CBO_Black eigengene module in prenatal fetal cortex as well as in the brain organoids' datasets. PCA was performed to calculate the module eigengene on the CBO dataset and then applied to BS prenatal cortex, as well as each of the other brain organoid datasets. Each dot represents a data point, while the line connects the median value for each postconceptional week (BS) or differentiation day (BO). The same analysis and visualization are applied to CBO_Brown and CBO_Blue (**B**, decreasing during differentiation) and CBO_Green and CBO_Red (**C**, nonmonotonic behaviour).

Supplementary Table 1

Differentially expressed genes with FDR < 0.05 and abs(logFC) > 1 from differential expression analyses.

Supplementary Table 2

Neurotransmission signature genes with the specification of their role in the relative neurotransmission system (Enzyme, receptor or transporter).

Supplementary Table 3

Results of Gene Ontology analyses.

Figure S1

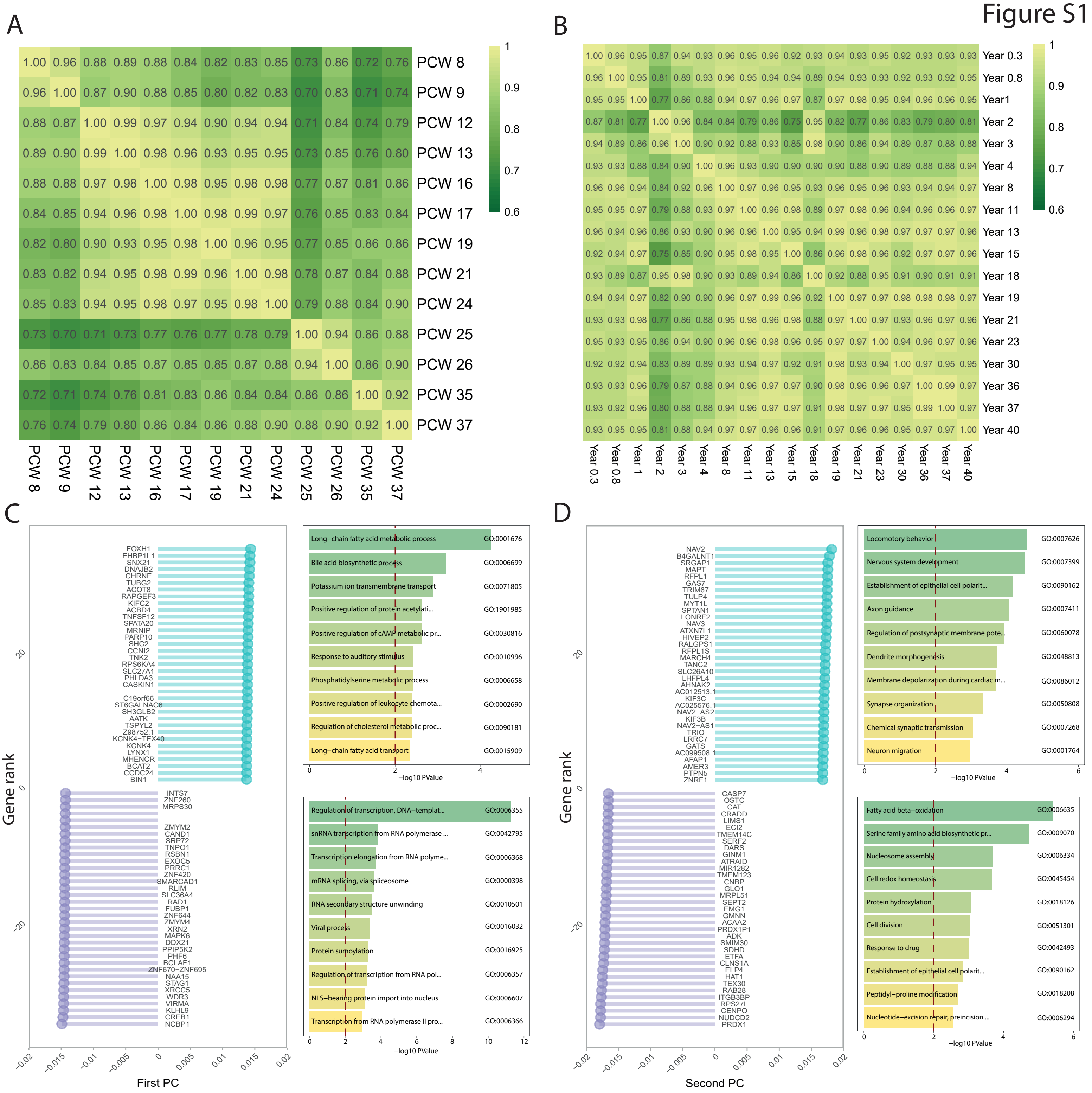
A
PCW 8 1.00 0.96 0.88 0.89 0.88 0.84 0.82 0.83 0.85 0.73 0.86 0.72 0.76
PCW 9 0.96 1.00 0.87 0.90 0.88 0.85 0.80 0.82 0.83 0.70 0.83 0.71 0.74
PCW 12 0.88 0.87 1.00 0.99 0.97 0.94 0.90 0.94 0.94 0.71 0.84 0.74 0.79
PCW 13 0.89 0.90 0.99 1.00 0.98 0.96 0.93 0.95 0.95 0.73 0.85 0.76 0.80
PCW 16 0.88 0.88 0.97 0.98 1.00 0.98 0.95 0.98 0.98 0.77 0.87 0.81 0.86
PCW 17 0.84 0.85 0.94 0.96 0.98 1.00 0.98 0.99 0.97 0.76 0.85 0.83 0.84
PCW 19 0.82 0.80 0.90 0.93 0.95 0.98 1.00 0.96 0.95 0.77 0.85 0.86 0.86
PCW 21 0.83 0.82 0.94 0.95 0.98 0.99 0.96 1.00 0.98 0.78 0.87 0.84 0.88
PCW 24 0.85 0.83 0.94 0.95 0.98 0.97 0.95 0.98 1.00 0.79 0.88 0.84 0.90
PCW 25 0.73 0.70 0.71 0.73 0.77 0.76 0.77 0.78 0.79 1.00 0.94 0.86 0.88
PCW 26 0.86 0.83 0.84 0.85 0.87 0.85 0.85 0.87 0.88 0.94 1.00 0.86 0.90
PCW 35 0.72 0.71 0.74 0.76 0.81 0.83 0.86 0.84 0.84 0.86 0.86 1.00 0.92
PCW 37 0.76 0.74 0.79 0.80 0.86 0.84 0.86 0.88 0.90 0.88 0.90 0.92 1.00
PCW 8 PCW 9 PCW 12 PCW 13 PCW 16 PCW 17 PCW 19 PCW 21 PCW 24 PCW 25 PCW 26 PCW 35 PCW 37
1 0.9 0.8 0.7 0.6
B
Year 0.3 Year 0.8 Year1 Year 2 Year 3 Year 4 Year 8 Year 11 Year 13 Year 15 Year 18 Year 19 Year 21 Year 23 Year 30 Year 36 Year 37 Year 40
1 0.9 0.8 0.7 0.6
C
Gene rank
First PC
FOXH1 EHBP1L1 SNX21 DNAJB2 CHRNE TUBG2 ACOT8 RAPGEF3 KIFC2 ACBD4 TNFSF12 SPATA20 MRNIP PARP10 SHC2 CCNI2 TNK2 RPS6KA4 SLC27A1 PHLDA3 CASKIN1 C19orf66 ST6GALNAC6 SH3GLB2 AATK TSPYL2 Z98752.1 KCNK4−TEX40 KCNK4 LYNX1 MHENCR BCAT2 CCDC24 BIN1
INTS7 ZNF260 MRPS30 ZMYM2 CAND1 SRP72 TNPO1 RSBN1 EXOC5 PRRC1 ZNF420 SMARCAD1 RLIM SLC36A4 RAD1 FUBP1 ZNF644 ZMYM4 XRN2 MAPK6 DDX21 PPIP5K2 PHF6 BCLAF1 ZNF670−ZNF695 NAA15 STAG1 XRCC5 WDR3 VIRMA KLHL9 CREB1 NCBP1
Long−chain fatty acid metabolic process GO:0001676
Bile acid biosynthetic process GO:0006699
Potassium ion transmembrane transport GO:0071805
Positive regulation of protein acetylati... GO:1901985
Positive regulation of cAMP metabolic pr... GO:0030816
Response to auditory stimulus GO:0010996
Phosphatidylserine metabolic process GO:0006658
Positive regulation of leukocyte chemota... GO:0002690
Regulation of cholesterol metabolic proc... GO:0090181
Long−chain fatty acid transport GO:0015909
−log10 PValue
Regulation of transcription, DNA−templat... GO:0006355
snRNA transcription from RNA polymerase ... GO:0042795
Transcription elongation from RNA polyme... GO:0006368
mRNA splicing, via spliceosome GO:0000398
RNA secondary structure unwinding GO:0010501
Viral process GO:0016032
Protein sumoylation GO:0016925
Regulation of transcription from RNA pol... GO:0006357
NLS−bearing protein import into nucleus GO:0006607
Transcription from RNA polymerase II pro... GO:0006366
−log10 PValue
D
Gene rank
Second PC
NAV2 B4GALNT1 SRGAP1 MAPT RFPL1 GAS7 TRIM67 TULP4 MYT1L SPTAN1 LONRF2 NAV3 ATXN7L1 HIVEP2 RALGPS1 RFPL1S MARCH4 TANC2 SLC26A10 LHFPL4 AHNAK2 AC012513.1 KIF3C AC025576.1 NAV2−AS2 TRIO KIF3B NAV2−AS1 LRRC7 GATS AC099508.1 AFAP1 AMER3 PTPN5 ZNRF1
CASP7 OSTC CAT CRADD LIMS1 ECI2 TMEM14C SERF2 DARS GINM1 ATRAID MIR1282 TMEM123 CNBP GLO1 MRPL51 SEPT2 EMG1 GMNN ACAA2 PRDX1P1 ADK SMIM30 SDHD ETFA CLNS1A ELP4 HAT1 TEX30 RAB28 ITGB3BP RPS27L CENPQ NUDCD2 PRDX1
Locomotory behavior GO:0007626
Nervous system development GO:0007399
Establishment of epithelial cell polarit... GO:0090162
Axon guidance GO:0007411
Regulation of postsynaptic membrane pote... GO:0060078
Dendrite morphogenesis GO:0048813
Membrane depolarization during cardiac m... GO:0086012
Synapse organization GO:0050808
Chemical synaptic transmission GO:0007268
Neuron migration GO:0001764
−log10 PValue
Fatty acid beta−oxidation GO:0006635
Serine family amino acid biosynthetic pr... GO:0009070
Nucleosome assembly GO:0006334
Cell redox homeostasis GO:0045454
Protein hydroxylation GO:0018126
Cell division GO:0051301
Response to drug GO:0042493
Establishment of epithelial cell polarit... GO:0090162
Peptidyl−proline modification GO:0018208
Nucleotide−excision repair, preincision ... GO:0006294
−log10 PValue

Figure S2

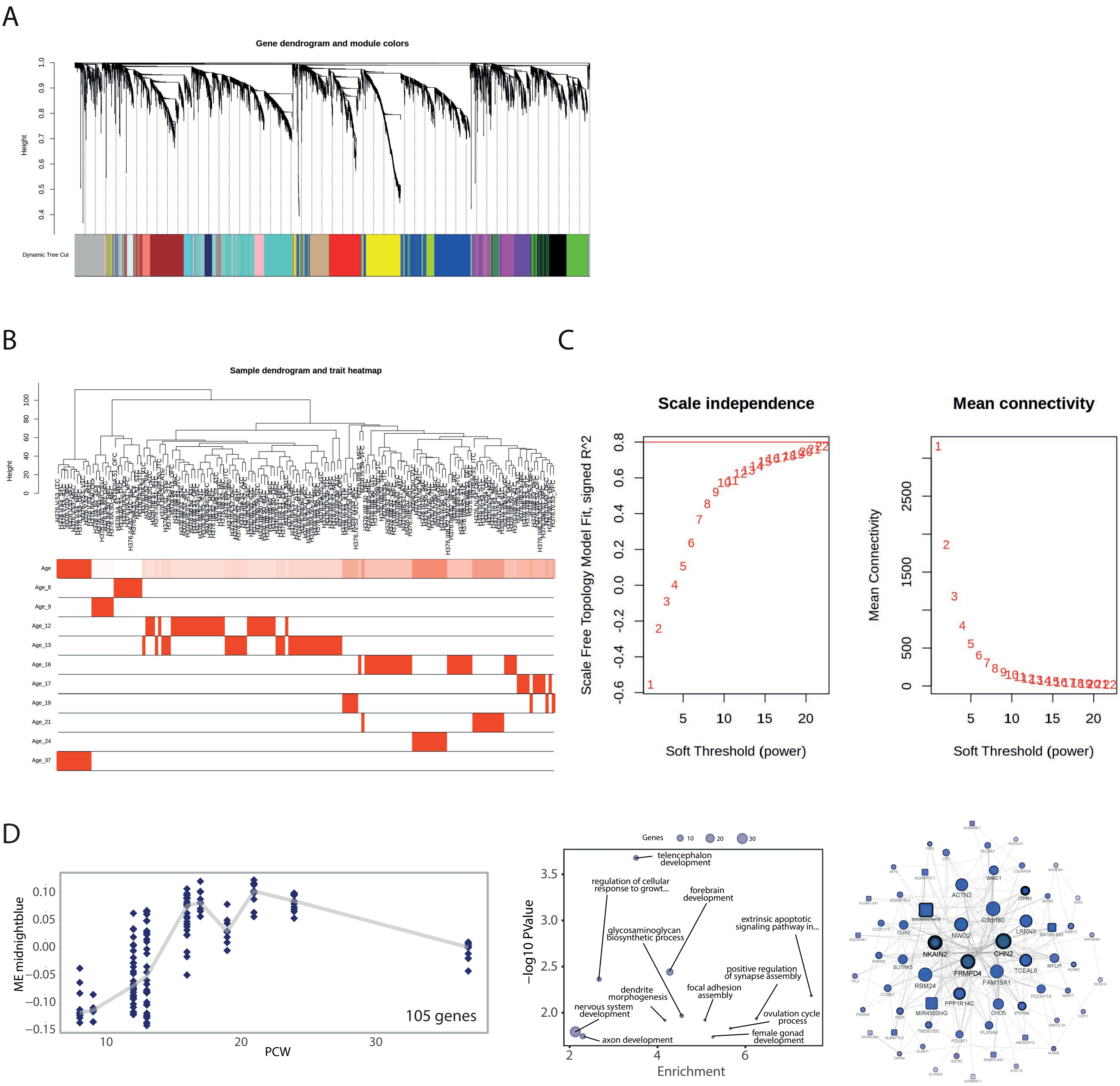
A
Gene dendrogram and module colors
Height
Dynamic Tree Cut
B
Sample dendrogram and trait heatmap
Height
Age
Age_8
Age_9
Age_12
Age_13
Age_16
Age_17
Age_19
Age_21
Age_24
Age_37
C
Scale independence
Scale Free Topology Model Fit, signed R^2
Soft Threshold (power)
Mean connectivity
Mean Connectivity
Soft Threshold (power)
D
ME midnightblue
PCW
105 genes
Genes
-log10 PValue
Enrichment
telencephalon development
regulation of cellular response to growt...
forebrain development
glycosaminoglycan biosynthetic process
extrinsic apoptotic signaling pathway in...
positive regulation of synapse assembly
dendrite morphogenesis
focal adhesion assembly
nervous system development
ovulation cycle process
female gonad development
axon development
ACTN2
WWC1
ITPR1
C3orf80
LRRN3
CUX2
NWD2
NKAIN2
CHN2
SLITRK5
FRMPD4
TCEAL6
MYLIP
RBM24
FAM19A1
PPP1R14C
MIR4500HG
CHD5
PTPRK

Figure S3

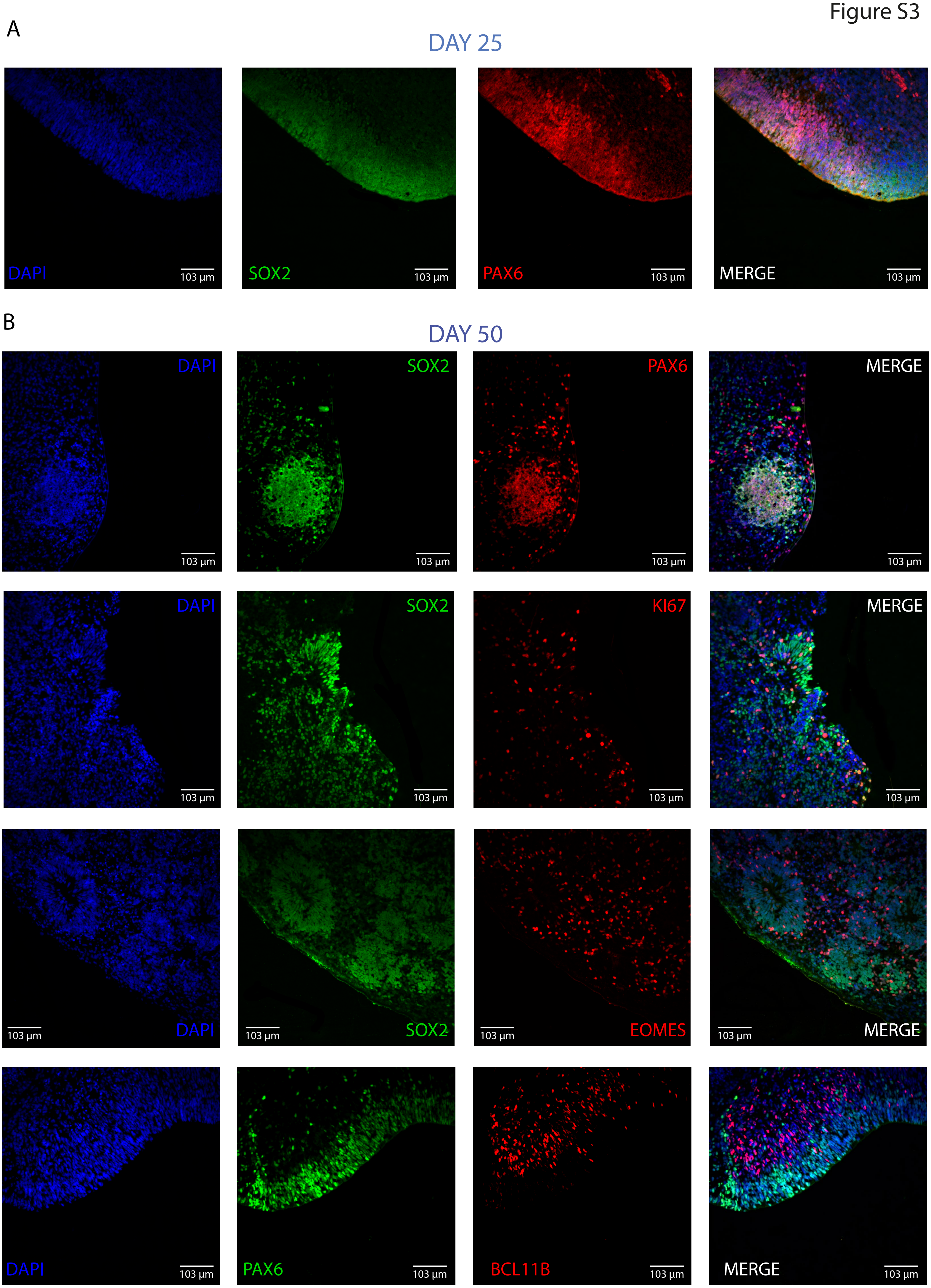
A
DAY 25
DAPI
103 µm
SOX2
103 µm
PAX6
103 µm
MERGE
103 µm
B
DAY 50
DAPI
SOX2
PAX6
MERGE
103 µm
DAPI
SOX2
KI67
MERGE
103 µm
DAPI
SOX2
EOMES
MERGE
103 µm
DAPI
PAX6
BCL11B
MERGE
103 µm

Figure S4

A

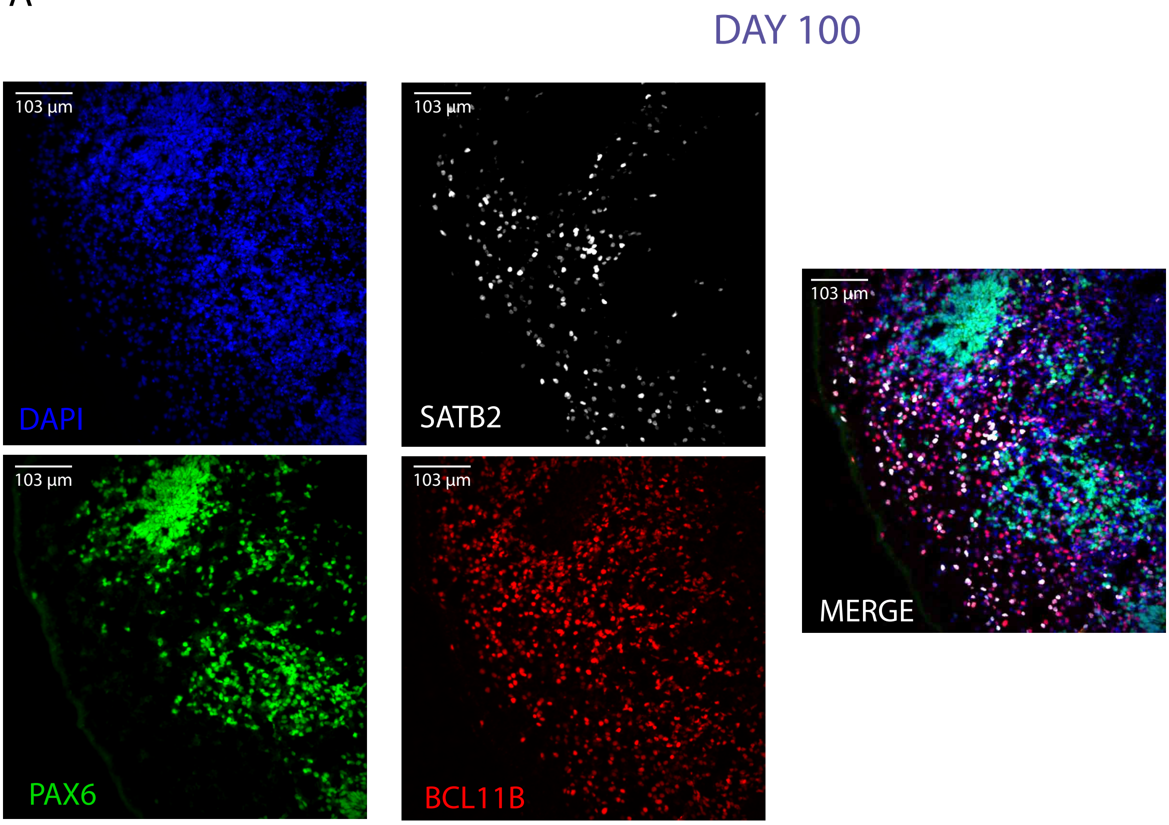
DAY 100
103 µm
DAPI
SATB2
PAX6
BCL11B
MERGE

B

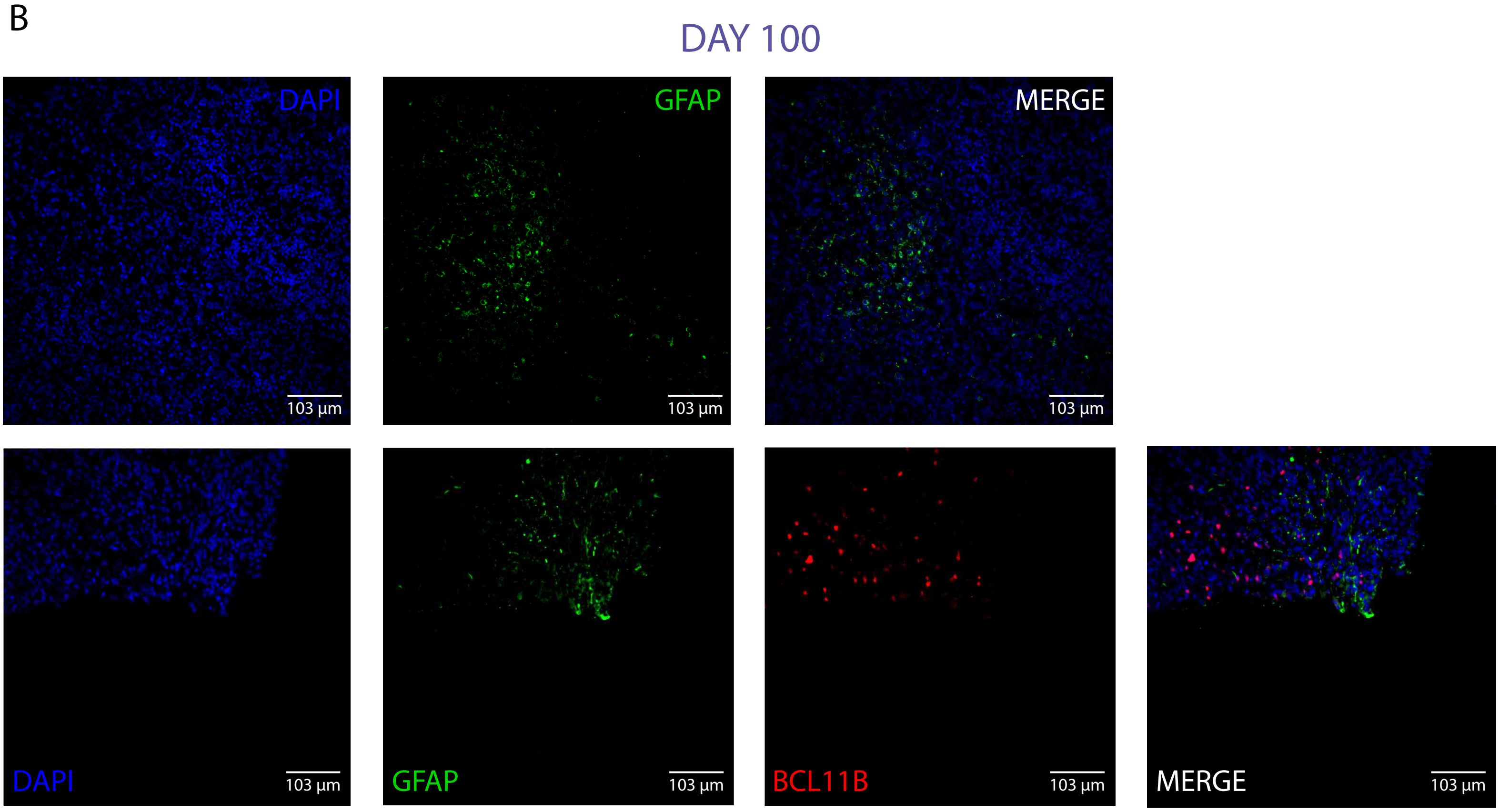
DAY 100
DAPI
GFAP
MERGE
BCL11B
103 µm

Figure S5

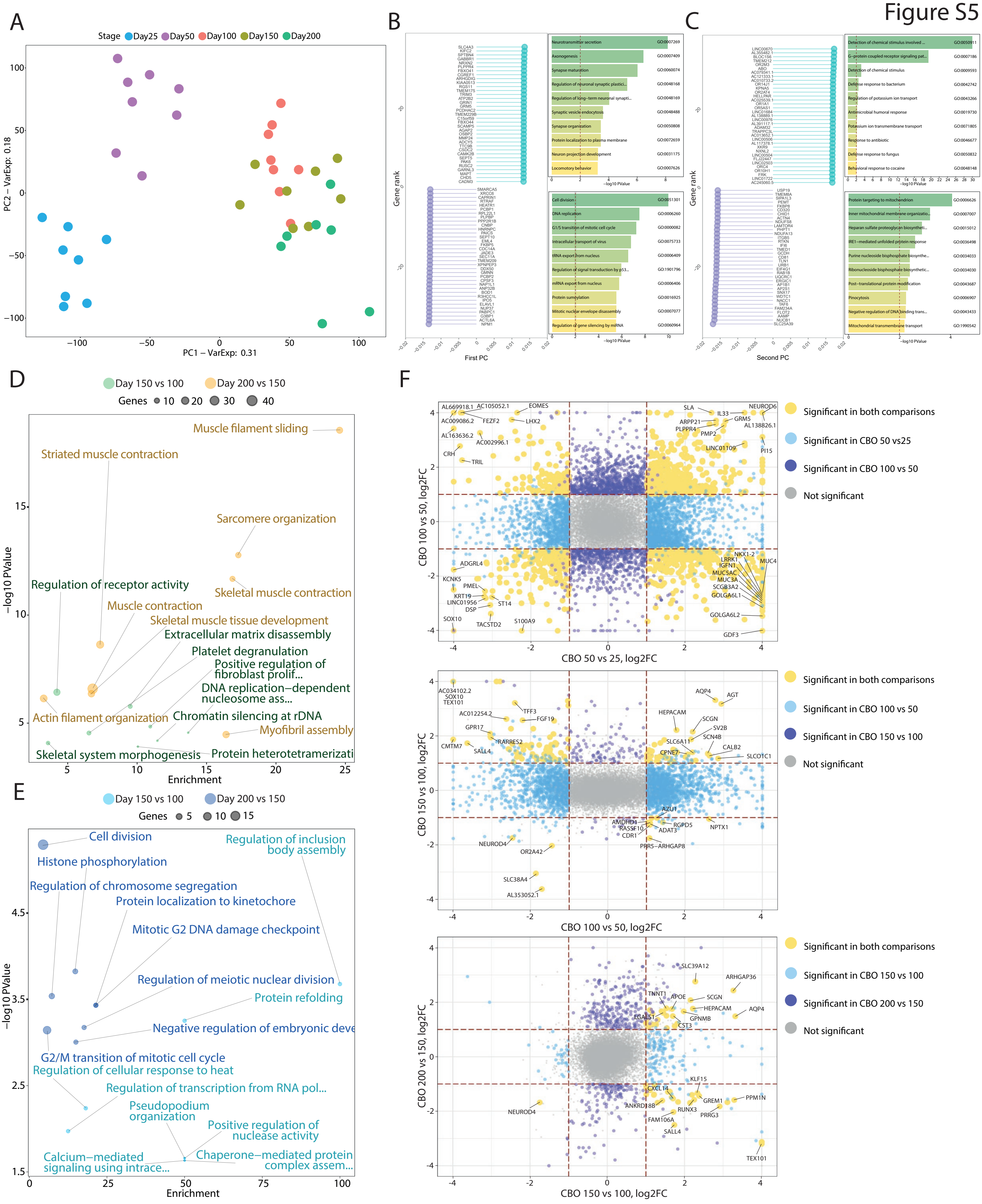
A
Stage Day25 Day50 Day100 Day150 Day200
PC1 – VarExp: 0.31
PC2 – VarExp: 0.18
B
First PC
Gene rank
C
Second PC
D
Day 150 vs 100
Day 200 vs 150
Genes 10 20 30 40
Muscle filament sliding
Striated muscle contraction
Sarcomere organization
Regulation of receptor activity
Skeletal muscle contraction
Muscle contraction
Skeletal muscle tissue development
Extracellular matrix disassembly
Platelet degranulation
Positive regulation of fibroblast prolif...
DNA replication−dependent nucleosome ass...
Actin filament organization
Chromatin silencing at rDNA
Myofibril assembly
Skeletal system morphogenesis
Protein heterotetramerizati
−log10 PValue
Enrichment
E
Day 150 vs 100
Day 200 vs 150
Genes 5 10 15
Cell division
Regulation of inclusion body assembly
Histone phosphorylation
Regulation of chromosome segregation
Protein localization to kinetochore
Mitotic G2 DNA damage checkpoint
Regulation of meiotic nuclear division
Protein refolding
Negative regulation of embryonic deve
G2/M transition of mitotic cell cycle
Regulation of cellular response to heat
Regulation of transcription from RNA pol...
Pseudopodium organization
Positive regulation of nuclease activity
Calcium−mediated signaling using intrace...
Chaperone−mediated protein complex assem...
−log10 PValue
Enrichment
F
Significant in both comparisons
Significant in CBO 50 vs25
Significant in CBO 100 vs 50
Not significant
CBO 100 vs 50, log2FC
CBO 50 vs 25, log2FC
Significant in both comparisons
Significant in CBO 100 vs 50
Significant in CBO 150 vs 100
Not significant
CBO 150 vs 100, log2FC
CBO 100 vs 50, log2FC
Significant in both comparisons
Significant in CBO 150 vs 100
Significant in CBO 200 vs 150
Not significant
CBO 200 vs 150, log2FC
CBO 150 vs 100, log2FC

Figure S6

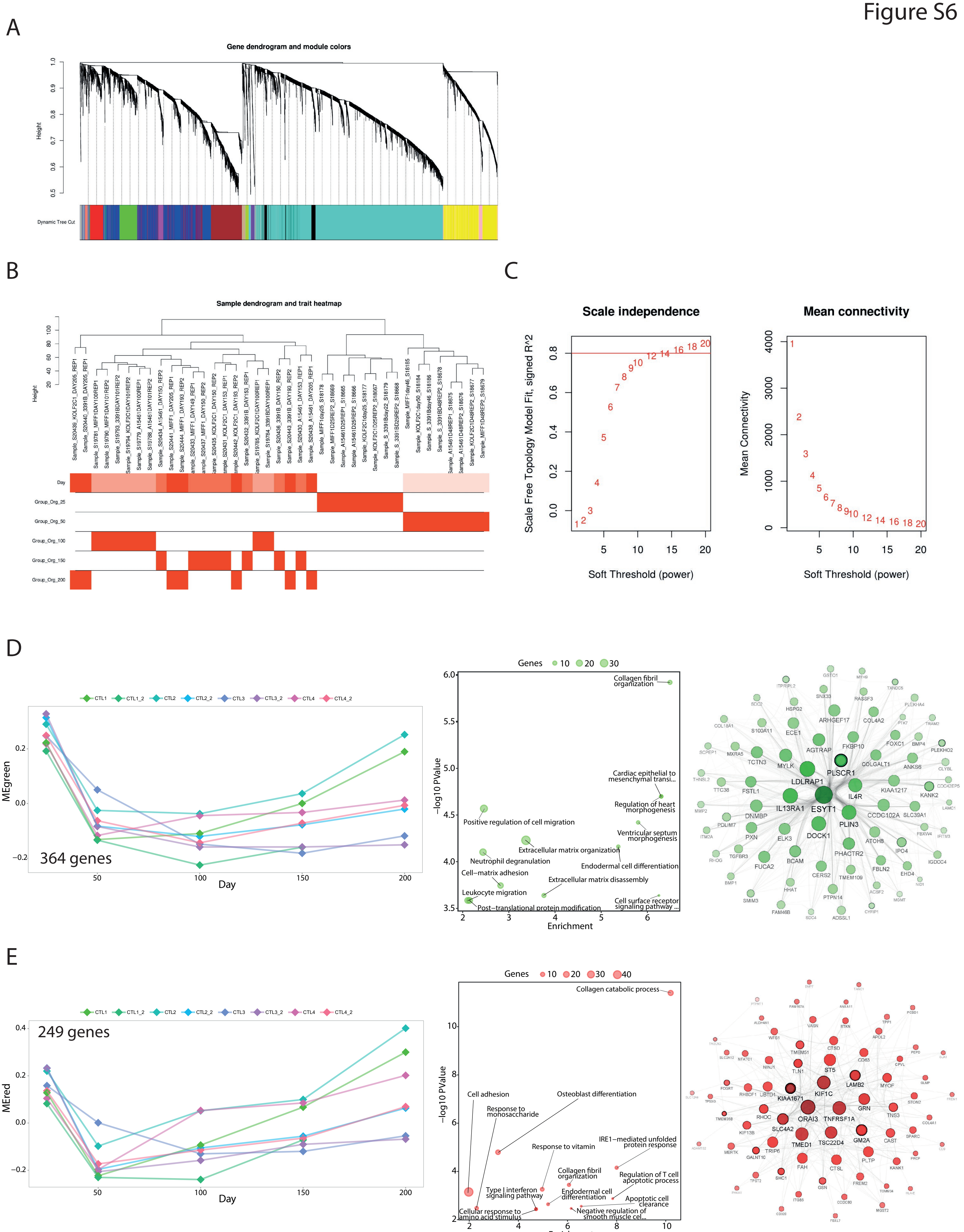
A
Gene dendrogram and module colors
Height
Dynamic Tree Cut
B
Sample dendrogram and trait heatmap
Day
Group_Org_25
Group_Org_50
Group_Org_100
Group_Org_150
Group_Org_200
C
Scale independence
Scale Free Topology Model Fit, signed R^2
Soft Threshold (power)
Mean connectivity
Mean Connectivity
Soft Threshold (power)
D
MEgreen
364 genes
Day
Genes 10 20 30
-log10 PValue
Enrichment
Collagen fibril organization
Cardiac epithelial to mesenchymal transi...
Regulation of heart morphogenesis
Positive regulation of cell migration
Ventricular septum morphogenesis
Extracellular matrix organization
Neutrophil degranulation
Endodermal cell differentiation
Cell−matrix adhesion
Extracellular matrix disassembly
Leukocyte migration
Cell surface receptor signaling pathway ...
Post−translational protein modification
E
MEred
249 genes
Day
Genes 10 20 30 40
Collagen catabolic process
Cell adhesion
Osteoblast differentiation
Response to monosaccharide
Response to vitamin
IRE1−mediated unfolded protein response
Collagen fibril organization
Regulation of T cell apoptotic process
Type I interferon signaling pathway
Endodermal cell differentiation
Apoptotic cell clearance
Cellular response to amino acid stimulus
Negative regulation of smooth muscle cel...
Enrichment

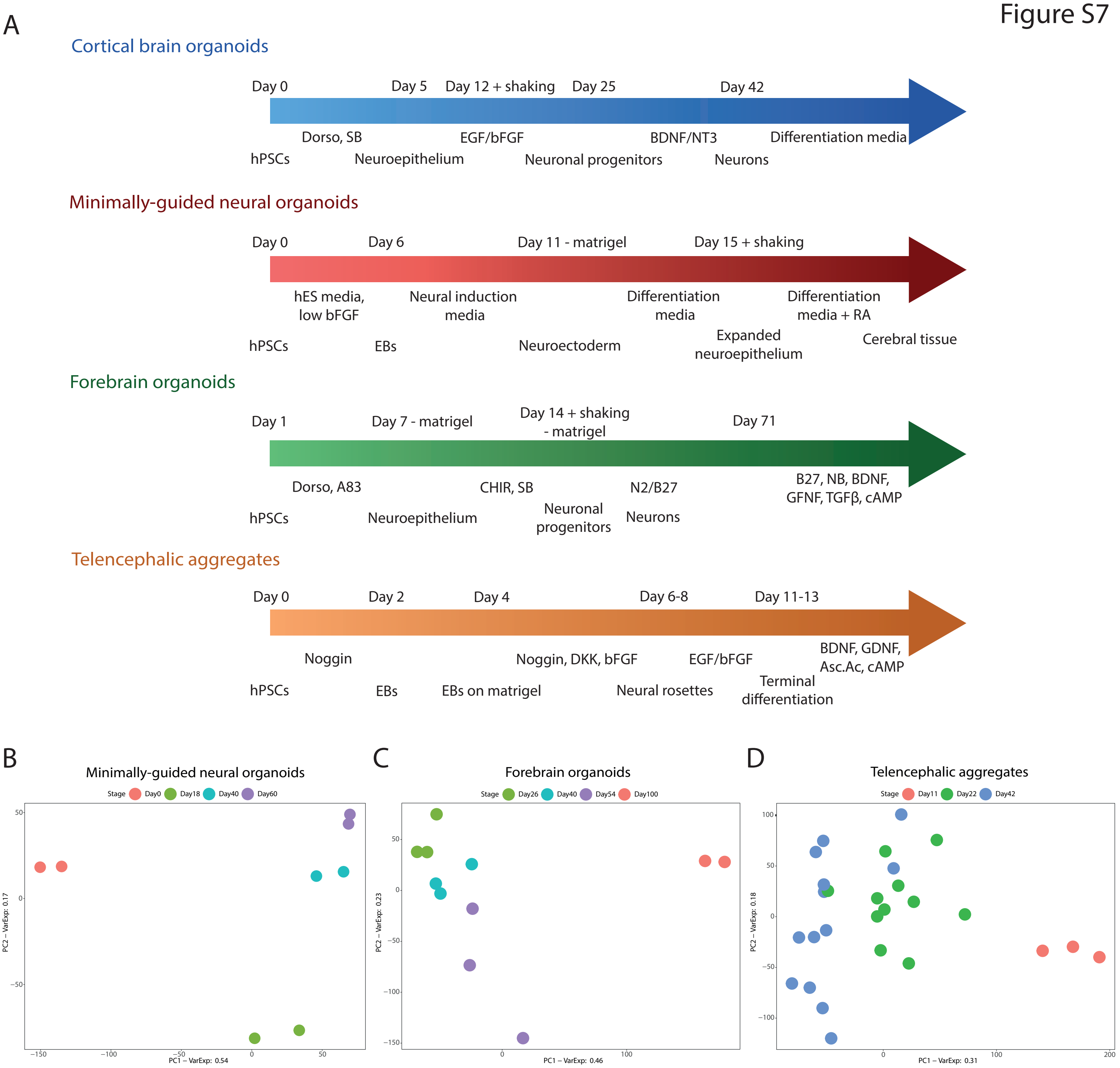
A
Cortical brain organoids
Day 0
Day 5
Day 12 + shaking
Day 25
Day 42
Dorso, SB
EGF/bFGF
BDNF/NT3
Differentiation media
hPSCs
Neuroepithelium
Neuronal progenitors
Neurons
Minimally-guided neural organoids
Day 0
Day 6
Day 11 - matrigel
Day 15 + shaking
hES media, low bFGF
Neural induction media
Differentiation media
Differentiation media + RA
hPSCs
EBs
Neuroectoderm
Expanded neuroepithelium
Cerebral tissue
Forebrain organoids
Day 1
Day 7 - matrigel
Day 14 + shaking - matrigel
Day 71
Dorso, A83
CHIR, SB
N2/B27
B27, NB, BDNF, GFNF, TGFβ, cAMP
hPSCs
Neuroepithelium
Neuronal progenitors
Neurons
Telencephalic aggregates
Day 0
Day 2
Day 4
Day 6-8
Day 11-13
Noggin
Noggin, DKK, bFGF
EGF/bFGF
BDNF, GDNF, Asc.Ac, cAMP
hPSCs
EBs
EBs on matrigel
Neural rosettes
Terminal differentiation
B
Minimally-guided neural organoids
Stage Day0 Day18 Day40 Day60
PC1 – VarExp: 0.54
PC2 – VarExp: 0.17
C
Forebrain organoids
Stage Day26 Day40 Day54 Day100
PC1 – VarExp: 0.46
PC2 – VarExp: 0.23
D
Telencephalic aggregates
Stage Day11 Day22 Day42
PC1 – VarExp: 0.31
PC2 – VarExp: 0.18

Figure S8

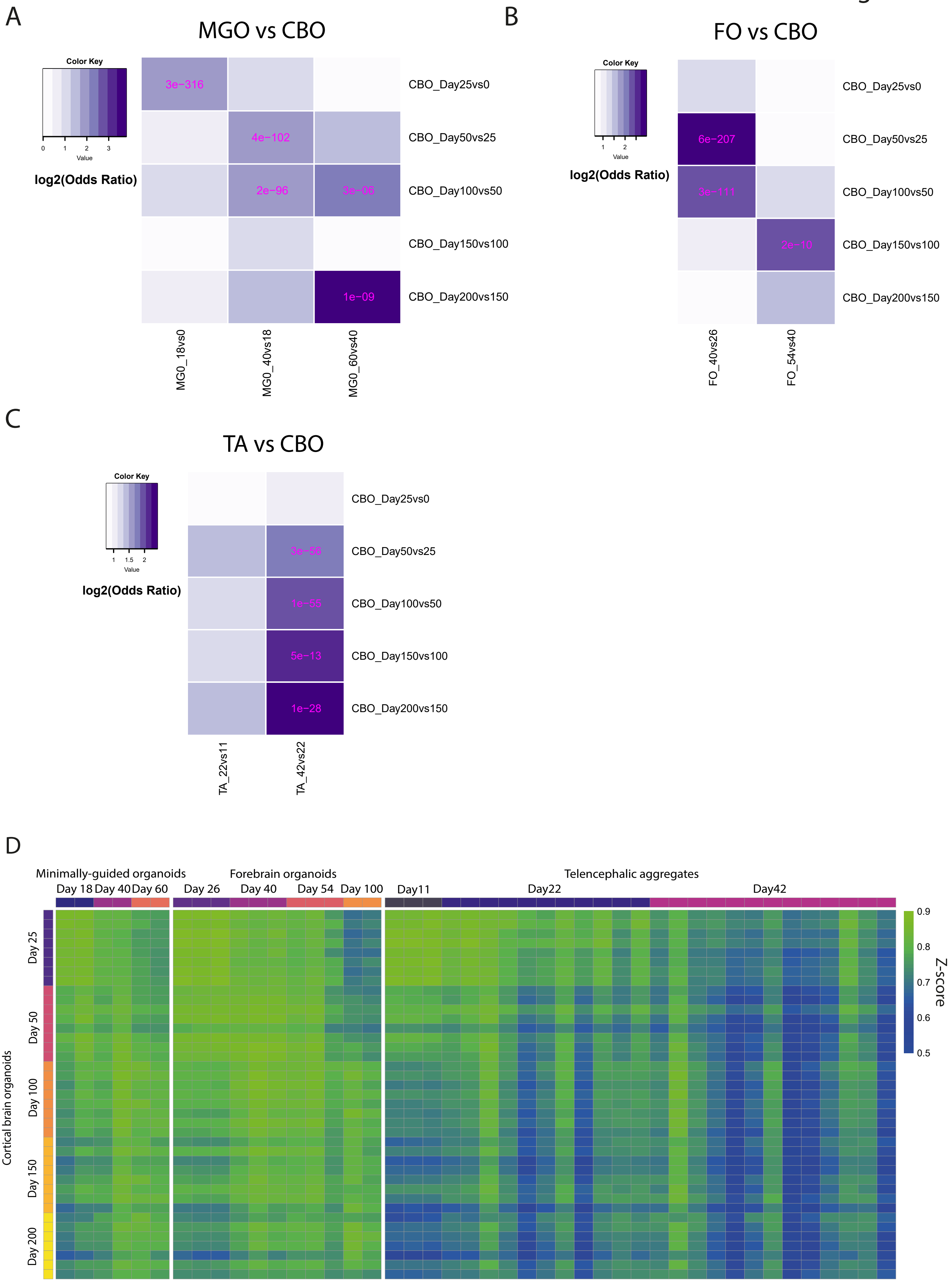
A
MGO vs CBO
Color Key
0 1 2 3
Value
log2(Odds Ratio)
3e−316
4e−102
2e−96
3e−06
1e−09
CBO_Day25vs0
CBO_Day50vs25
CBO_Day100vs50
CBO_Day150vs100
CBO_Day200vs150
MGO_18vs0
MGO_40vs18
MGO_60vs40
B
FO vs CBO
Color Key
1 2
Value
log2(Odds Ratio)
6e−207
3e−111
2e−10
CBO_Day25vs0
CBO_Day50vs25
CBO_Day100vs50
CBO_Day150vs100
CBO_Day200vs150
FO_40vs26
FO_54vs40
C
TA vs CBO
Color Key
1 1.5 2
Value
log2(Odds Ratio)
3e−56
1e−55
5e−13
1e−28
CBO_Day25vs0
CBO_Day50vs25
CBO_Day100vs50
CBO_Day150vs100
CBO_Day200vs150
TA_22vs11
TA_42vs22
D
Minimally-guided organoids
Forebrain organoids
Telencephalic aggregates
Day 18
Day 40
Day 60
Day 26
Day 40
Day 54
Day 100
Day11
Day22
Day42
Day 25
Day 50
Day 100
Day 150
Day 200
Cortical brain organoids
0.9
0.8
0.7
0.6
0.5
Z-score

Figure S9

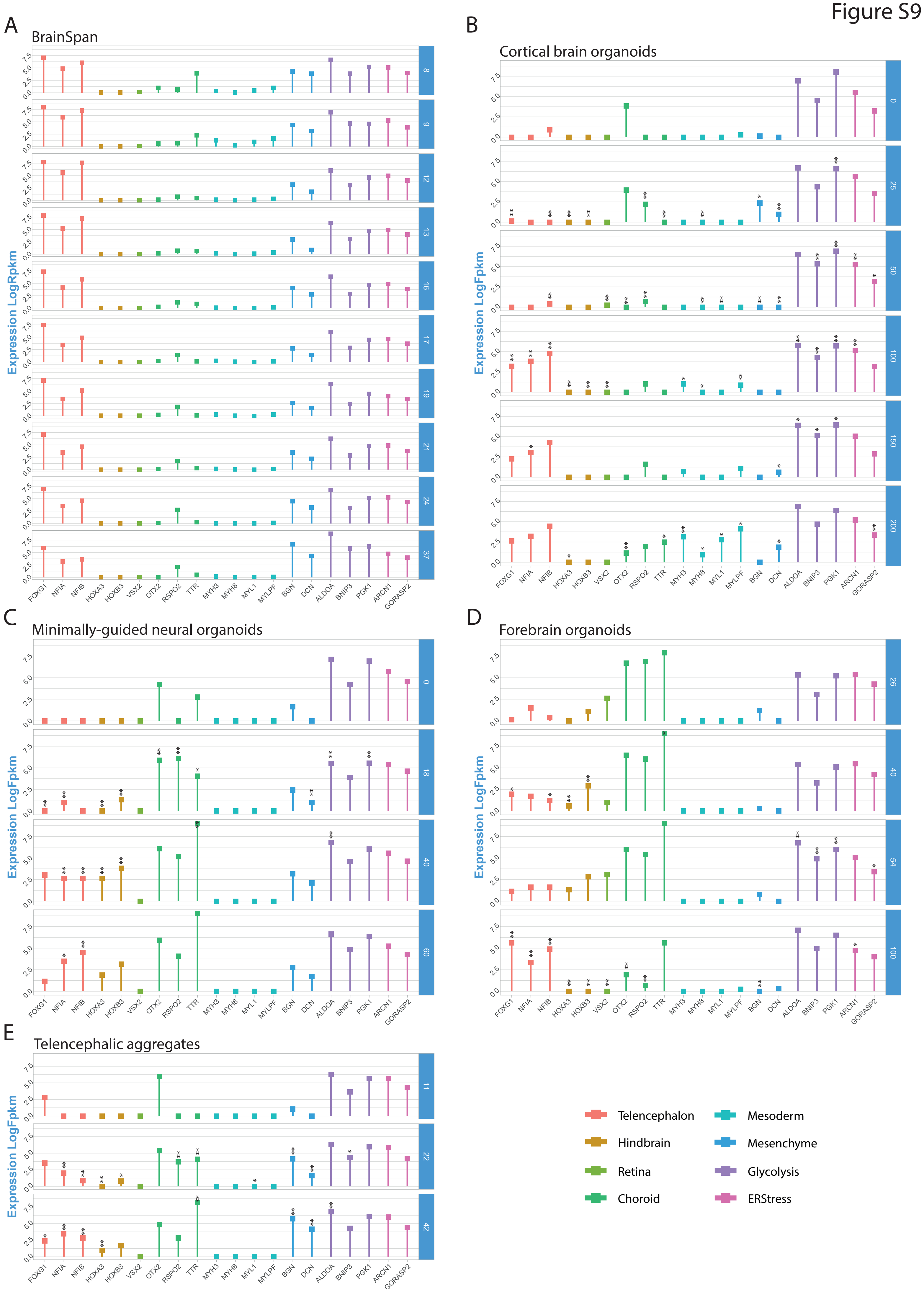
A
BrainSpan
Expression LogRpkm
8
9
12
13
16
17
19
21
24
37
B
Cortical brain organoids
Expression LogFpkm
0
25
50
100
150
200
C
Minimally-guided neural organoids
Expression LogFpkm
0
18
40
60
D
Forebrain organoids
Expression LogFpkm
26
40
54
100
E
Telencephalic aggregates
Expression LogFpkm
11
22
42
0.0
2.5
5.0
7.5
FOXG1
NFIA
NFIB
HOXA3
HOXB3
VSX2
OTX2
RSPO2
TTR
MYH3
MYH8
MYL1
MYLPF
BGN
DCN
ALDOA
BNIP3
PGK1
ARCN1
GORASP2
Telencephalon
Hindbrain
Retina
Choroid
Mesoderm
Mesenchyme
Glycolysis
ERStress

Figure S10

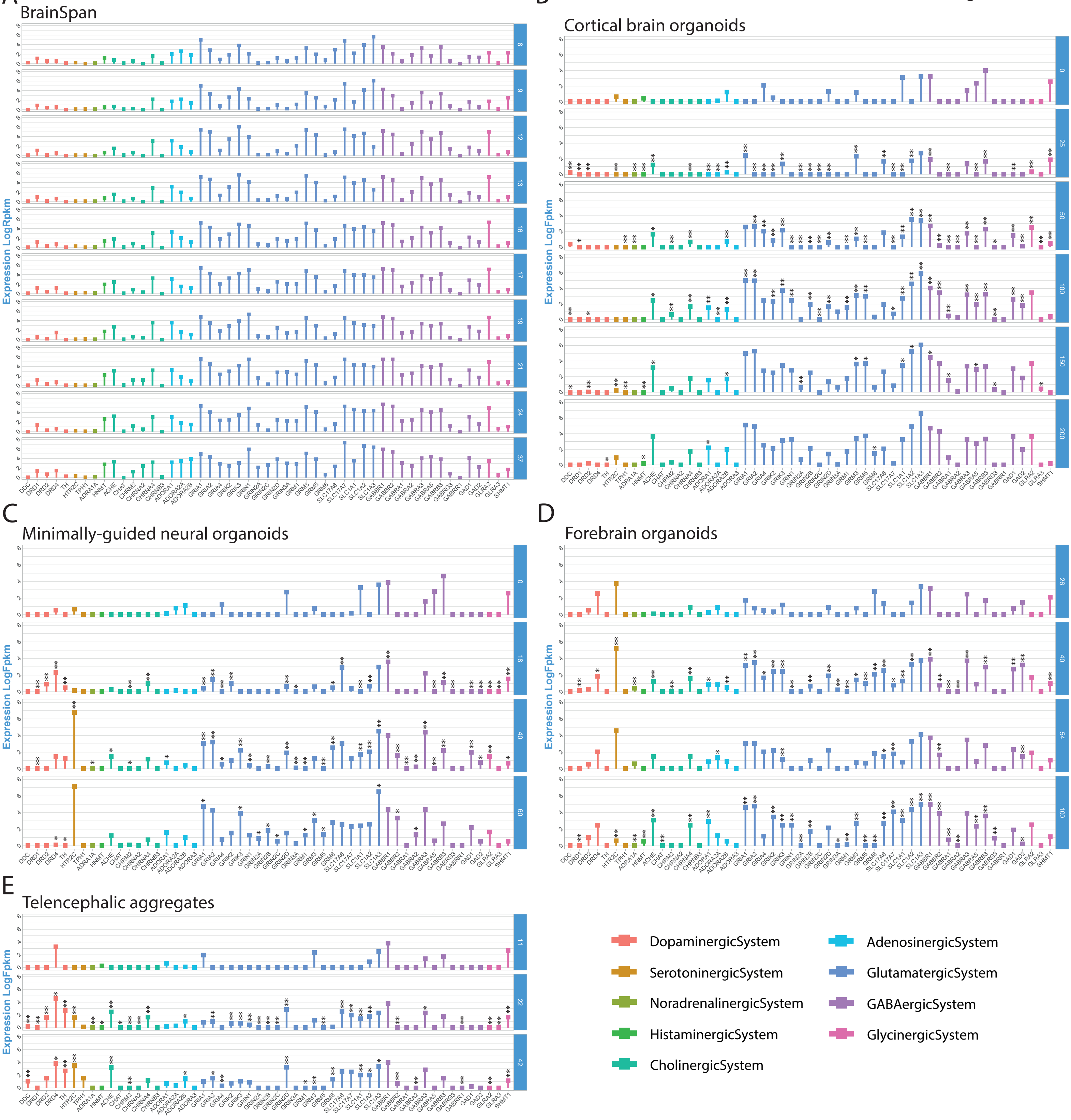
A
BrainSpan
Expression LogRpkm
B
Cortical brain organoids
Expression LogFpkm
C
Minimally-guided neural organoids
Expression LogFpkm
D
Forebrain organoids
Expression LogFpkm
E
Telencephalic aggregates
Expression LogFpkm
DopaminergicSystem
SerotoninergicSystem
NoradrenalinergicSystem
HistaminergicSystem
CholinergicSystem
AdenosinergicSystem
GlutamatergicSystem
GABAergicSystem
GlycinergicSystem

Figure S11

A

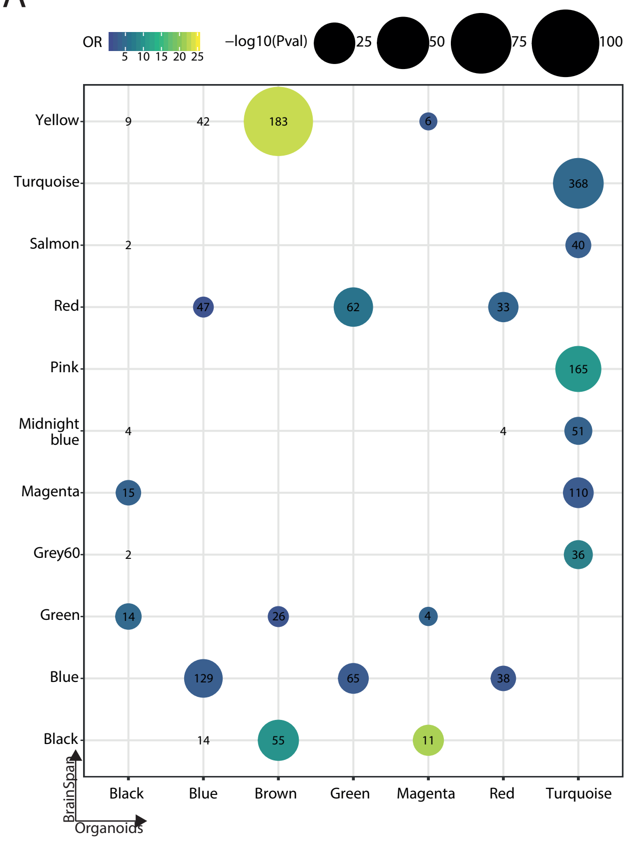

OR
5 10 15 20 25
−log10(Pval)
25
50
75
100
Yellow
Turquoise
Salmon
Red
Pink
Midnight blue
Magenta
Grey60
Green
Blue
Black
9
42
183
6
368
2
40
47
62
33
165
4
4
51
15
110
2
36
14
26
4
129
65
38
14
55
11
Black
Blue
Brown
Green
Magenta
Red
Turquoise
BrainSpan
Organoids

Figure S12

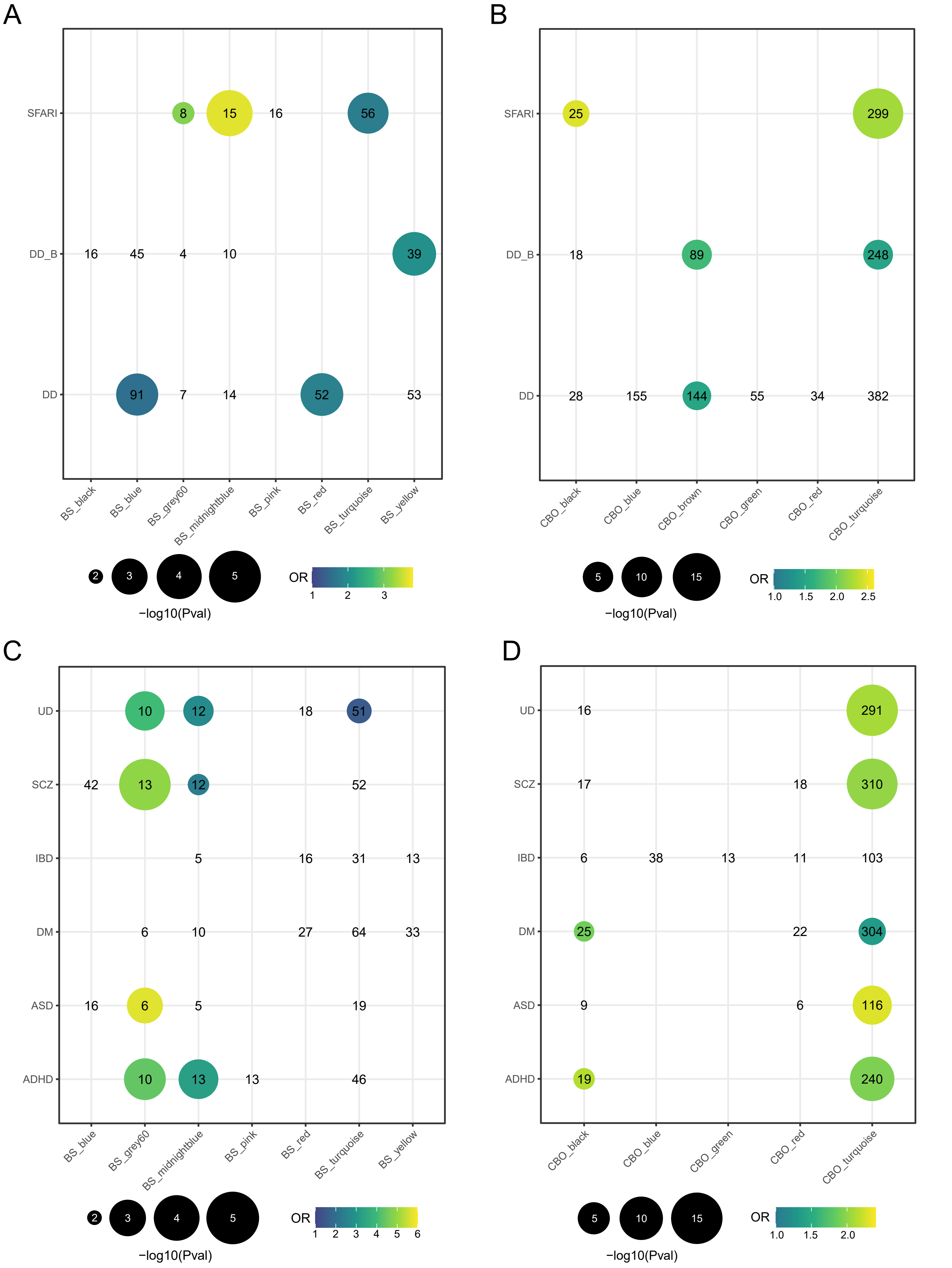
A
SFARI
DD_B
DD
8
15
16
56
16
45
4
10
39
91
7
14
52
53
BS_black
BS_blue
BS_grey60
BS_midnightblue
BS_pink
BS_red
BS_turquoise
BS_yellow
−log10(Pval)
OR
B
SFARI
DD_B
DD
25
299
18
89
248
28
155
144
55
34
382
CBO_black
CBO_blue
CBO_brown
CBO_green
CBO_red
CBO_turquoise
−log10(Pval)
OR
C
UD
SCZ
IBD
DM
ASD
ADHD
10
12
18
51
42
13
12
52
5
16
31
13
6
10
27
64
33
16
6
5
19
10
13
13
46
BS_blue
BS_grey60
BS_midnightblue
BS_pink
BS_red
BS_turquoise
BS_yellow
−log10(Pval)
OR
D
UD
SCZ
IBD
DM
ASD
ADHD
16
291
17
18
310
6
38
13
11
103
25
22
304
9
6
116
19
240
CBO_black
CBO_blue
CBO_green
CBO_red
CBO_turquoise
−log10(Pval)
OR

Figure S13

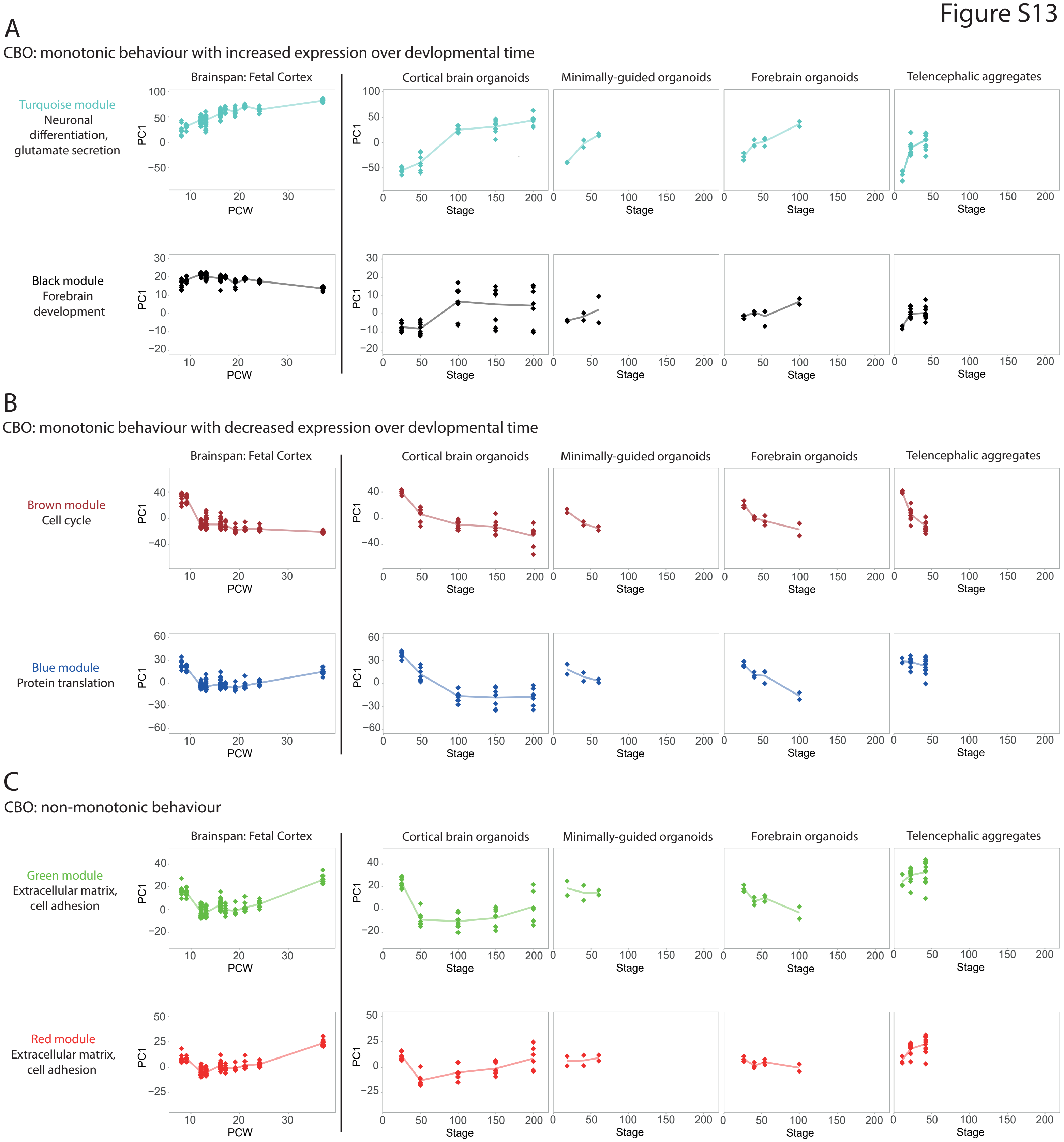
A
CBO: monotonic behaviour with increased expression over devlopmental time
Brainspan: Fetal Cortex
Cortical brain organoids
Minimally-guided organoids
Forebrain organoids
Telencephalic aggregates
Turquoise module
Neuronal differentiation, glutamate secretion
Black module
Forebrain development
PC1
PCW
Stage
B
CBO: monotonic behaviour with decreased expression over devlopmental time
Brown module
Cell cycle
Blue module
Protein translation
C
CBO: non-monotonic behaviour
Green module
Extracellular matrix, cell adhesion
Red module
Extracellular matrix, cell adhesion
