# Supplementary material for: Benchmarking brain organoid recapitulation of fetal corticogenesis
Source: Transl Psychiatry. 2022 Dec 20;12:520. doi: 10.1038/s41398-022-02279-0 (PMC9767930; doi:10.1038/s41398-022-02279-0)
Supplement: Supplementary file 5 — Supplementary file 1 [file 41398_2022_2279_MOESM5_ESM.html]

Brainspan WGCNA: Module Characterization


Code 

- Show All Code
- Hide All Code

# Brainspan WGCNA: Module Characterization

**WGCNA analysis on Brainspan fetal cortex: module characterization.**

## 1. SetUp

```
library(viridis)
## Loading required package: viridisLite
library(pheatmap)
library(DT)
library(tidyr)
library(dplyr)
## 
## Attaching package: 'dplyr'
## The following objects are masked from 'package:stats':
## 
##     filter, lag
## The following objects are masked from 'package:base':
## 
##     intersect, setdiff, setequal, union
library(ggplot2)
library(ggiraph)
library(WGCNA)
## Loading required package: dynamicTreeCut
## Loading required package: fastcluster
## 
## Attaching package: 'fastcluster'
## The following object is masked from 'package:stats':
## 
##     hclust
## 
## ==========================================================================
## *
## *  Package WGCNA 1.64.1 loaded.
## *
## *    Important note: It appears that your system supports multi-threading,
## *    but it is not enabled within WGCNA in R. 
## *    To allow multi-threading within WGCNA with all available cores, use 
## *
## *          allowWGCNAThreads()
## *
## *    within R. Use disableWGCNAThreads() to disable threading if necessary.
## *    Alternatively, set the following environment variable on your system:
## *
## *          ALLOW_WGCNA_THREADS=<number_of_processors>
## *
## *    for example 
## *
## *          ALLOW_WGCNA_THREADS=40
## *
## *    To set the environment variable in linux bash shell, type 
## *
## *           export ALLOW_WGCNA_THREADS=40
## *
## *     before running R. Other operating systems or shells will
## *     have a similar command to achieve the same aim.
## *
## ==========================================================================
## 
## Attaching package: 'WGCNA'
## The following object is masked from 'package:stats':
## 
##     cor
```

```
options(stringsAsFactors = FALSE)
```

```
#Helper file already used for the WGCNA on organoids
source('../../6_WGCNA/WGCNAHelper.R')
```

---

## 2. Function tailoring

```
ribbonME <- function(METraits=METraits, module=module, ascisse='Age', title=NULL, Shape=18, DotSize=6.5) {
  
  # Arguments:
  # I. METraits: data frame specifying the module eigengene and the continuous trait of interest (Day); a Rep column must specify the sample grouping. Additional columns can be present.
  # II. Module: string indicating the module to be represented
  # III. title: plot title.If null, a title with the specified module is generated
  # Dependencies: ggplot2
  
  # 1. Setting of plot title
  if (is.null(title)) {
    title <- paste0('Module Eigengene for ', toupper(module), ' Module')
  }  
  
  # 2. Ribbonplot representing module eigengene behaviour through day
  ggplot(data=METraits, aes(x=!!sym(ascisse), y=!!sym(paste0('ME', module)))) +
    geom_point(stat='identity', size=DotSize, shape=Shape, colour=module) +
    stat_summary(fun.y=median, colour='grey', geom='line', size=3.5, alpha=0.55) +
    ggtitle(title) + 
    theme_light()  +
    theme(plot.title = element_text(face='bold', colour='darkred', size=13, hjust=0.5)) 
    }
```

```
heatmapGeneModule <- function(data=LogMatrixSel, module=module, assignment=ModuleAssignment, title=NULL, annotation_col=NA, annotation_colors=NA, annotation_row=NA, cluster_rows=FALSE, cluster_cols=FALSE, scale='row', display_numbers=TRUE, display_genes=FALSE) {

  # Arguments: 
    # I. data: expression levels (as calculated by DESeq2), selected and transposed as required for WGCNA input 
    # II. module: 
    # III. assignment  
    # IV. title: string that will be incorporated in plot title
  
  # Dependencies: pheatmap, viridis

  # 1. Setting of plot title
  if (is.null(title)) {
    title <- paste0('Log Counts for ', toupper(module), ' Genes')
  }  
  
  # 2. Data selection
  Levels <- t(data[,assignment$Module==module])
  if(!identical(dimnames(data)[[2]], ModuleAssignment$Gene)){
    stop('Inconsistency between expression data and module assignment')
  }
    
  
  # 3. Color palette: 
  if (scale=='none'){
    MyPalette <- colorRampPalette(c('gray95', 'royalblue1'))(100)
  } else {
    MyPalette <-  viridis(100)
  }
  
  # 3. Heatmap
  
  pheatmap(data.frame(Levels), cluster_rows=cluster_rows, cluster_cols=cluster_cols, 
           clustering_method='average', display_numbers=display_numbers, col=MyPalette, scale=scale,
           annotation_col=annotation_col, annotation_row=annotation_row, annotation_colors=annotation_colors, 
           main=title, fontsize_col=6, fontsize_row=6, show_rownames=display_genes)
  
}
```

## 3. Data Upload

### 3.1 Gene Metadata

Duplicated Ensembl Gene Code are eliminated.

```
GeneInfo <- readRDS('/media/data/Organoids/NeurodevDatasets/4_DataExploration/AnnotationGenecode/Output/OutputAnnotation.rds')
GeneMeta <- GeneInfo$AnnotationDF %>% dplyr::select(1:5)
dim(GeneMeta)
## [1] 58288     5

table(duplicated(GeneMeta$EnsGene))
## 
## FALSE  TRUE 
## 58243    45

GeneMeta <- dplyr::filter(GeneMeta, ! duplicated(GeneMeta$EnsGene))
table(duplicated(GeneMeta$EnsGene))
## 
## FALSE 
## 58243
dim(GeneMeta)
## [1] 58243     5
```

### 3.2 Objects generated from the first step

```
load('/media/data/Organoids/NeurodevDatasets//8_Brainspan/E_WGCNA/1_NetworkGeneration/NetworkGeneration.RData')
```

- adjacency: adjacency matrix
- dissTOM: TOM distance matrix
- DummyTraits: phenotypic traits
- ModuleAssignment: assignment for each gene to a module
- MEs: module eigengenes
- LogMatrixSel: selected, transformed, transposed expression matrix

Heatmap Annotation

```
HeatAnno <- DummyTraits %>% dplyr::select(6, 8:17)
HeatAnno[, -1] <- sapply(HeatAnno[, -1], as.character)

row.names(HeatAnno) <- sub('-', '.', DummyTraits$ID)

HeatCols <- list()
HeatCols$Age_8 <- c('1'='blue', '0'="grey")
HeatCols$Age_9 <- c('1'='blue', '0'="grey")
HeatCols$Age_12 <- c('1'='blue', '0'="grey")
HeatCols$Age_13 <- c('1'='blue', '0'="grey")
HeatCols$Age_16 <- c('1'='blue', '0'="grey")
HeatCols$Age_17 <- c('1'='blue', '0'="grey")
HeatCols$Age_19 <- c('1'='blue', '0'="grey")
HeatCols$Age_21 <- c('1'='blue', '0'="grey")
HeatCols$Age_24 <- c('1'='blue', '0'="grey")
HeatCols$Age_37 <- c('1'='blue', '0'="grey")
```

---

## 4. Module correlation with phenotypic traits

### 4.1 Correlation between module eigengenes and phenotypic traits

```
# Sub-structure
for(level in unique(factor(DummyTraits$Str))){
  DummyTraits[paste("Str", level, sep = "_")] <- ifelse(DummyTraits$Str == level, 1, 0)
}
```

Calculate the correlation with modules and the phenotypic traits using spearman correlation

```
nGenes <- ncol(LogMatrixSel) 
nSamples <- nrow(LogMatrixSel)

moduleTraitCor <- WGCNA::cor(MEs, DummyTraits[, c(6, 8:32)], use = 'p', method='spearman')
moduleTraitPvalue <- WGCNA::corPvalueStudent(moduleTraitCor, nSamples)
```

### 4.2 Visualization of the correlation

**Complete heatmap**

```
textMatrix <- paste(signif(moduleTraitCor,2), '\n(', signif(moduleTraitPvalue, 1), ')', sep = '')
dim(textMatrix) <- dim(moduleTraitCor)
WGCNA::labeledHeatmap(Matrix=moduleTraitCor, xLabels=names(DummyTraits[, c(6, 8:32)]), yLabels=names(MEs), ySymbols=names(MEs), colorLabels=FALSE, colors=viridis(50)[10:50], textMatrix = textMatrix, setStdMargins = FALSE, cex.text = 0.5, zlim = c(-1,1), main = paste('Module-trait relationships'))
```

**Selected heatmap**

```
textMatrix <- paste(signif(moduleTraitCor[, 1:11],2), '\n(', signif(moduleTraitPvalue[, 1:11], 1), ')', sep = '')
dim(textMatrix) <- dim(moduleTraitCor[, 1:11])
WGCNA::labeledHeatmap(Matrix=moduleTraitCor[, 1:11], xLabels=names(DummyTraits[, c(6, 8:17)]), yLabels=names(MEs), ySymbols=names(MEs), colorLabels=FALSE, colors=viridis(50)[10:50], textMatrix=textMatrix, setStdMargins = FALSE, cex.text = 0.5, zlim = c(-1,1), main = paste('Module-trait relationships'))
```

**Overall Positive correlation with developmental day:**

- **Turquoise, Pink**: highest positive correlation with PCW as continuous variable.
- **Salmon, Grey60, NightBlue**: positive correlation with PCW; especially low till PCW13
- **Brown and Tan**: mild positive correlation. Brown especially low at PCW 12-13; tan similar but less marked.

**Overall Negative correlation with developmental day:**

- **Black, Green**: very strong negative correlation with continuous PCW. Green top positive correlation at PCW 13.
- **Yellow**: negative correlation with continuous PCW; strong low values at first weeks.
- **Purple**: negative correlation with continuous PCW; low correlation with first and latest PCW.

**No strong correlation with developmental day:**

- **Blue, Cyan**: Start positive, strong decrease 12-13, increase again
- **Red**: negative at PCW 13
- **Magenta**: positive at PCW 12-13 \***GreenYellow, LightCyan**

---

## 5. Gene-related metrics: Module Membership and Connectivity

### 5.1 Calculate Gene Module Membership

The **Gene Module Membership** is a metrics that quantify the membership of each gene to all the identified modules.

Calculation of the correlation coefficient:

```
# Calculation of correlation coefficient and p-value (approximized by Student asymptotic p-value)
Cor <- corAndPvalue(LogMatrixSel, MEs, use='p',  method='spearman')

CorCoeff <- data.frame(Cor$cor)
names(CorCoeff) <- paste0('MM', substring(names(CorCoeff), 3))
CorCoeff$Gene <- row.names(Cor$cor)

CorP <- data.frame(Cor$p)
names(CorP) <- paste0('p_MM', substring(names(CorP), 3))
CorP$Gene <- row.names(Cor$p)

# Generation of data frame containing relevant information
GeneMM <- dplyr::inner_join(CorCoeff, CorP, by='Gene') %>% 
  dplyr::inner_join(ModuleAssignment, by='Gene') %>%
  dplyr::left_join(GeneMeta, by=c('Gene'='EnsGene')) %>% dplyr::select(19, 38, 1:18, 20:37, 16, 30, 40:42)

dim(GeneMM)
## [1] 5889   41
```

### 5.2 Connectivity Measures

**Intramodular connectivity (kWithin)** calculates for each gene the connectivity with other genes belonging to the same module. Other measures that are calculated are the total connectivity (kTotal), the connectivity outside the module (kOut) and the difference between intra-modular and extra-modular connectivity (kDiff). All the metrics calculated at the gene level (module membership and connectivity) are stored in the **GeneMetrics** data frame.

```
GeneCon <- intramodularConnectivity(adjacency, colors=ModuleAssignment$Module)
GeneCon$Gene <- row.names(GeneCon)
GeneMetrics <- dplyr::inner_join(GeneMM, GeneCon, by='Gene')
```

---

## 6. Turquoise Module

```
ModName <- 'turquoise'
```

TURQUOISE module is composed by 782 genes.

**BOXPLOT**: distribution of TURQUOISE module membership stratified for module assignment.

```
boxMM(GeneMM=GeneMetrics, module=ModName, title=NULL)
```

**RIBBON of Module Eigengene**: behavior of module eigengene through time-points and samples.

```
METraits <- dplyr::mutate(MEs, Sample=row.names(LogMatrixSel)) %>% inner_join(DummyTraits, by=c('Sample'='ID')) 

ribbonME(METraits=METraits, module=ModName, ascisse='Age', title=NULL)
```

**HEATMAP**: gene expression values (log transformed read counts) for the genes belonging to the TURQUOISE module.

```
heatmapGeneModule(data=LogMatrixSel, module=ModName, assignment=ModuleAssignment, title=NULL, annotation_col=HeatAnno, annotation_colors=HeatCols, annotation_row=NA, cluster_rows=TRUE, cluster_cols=TRUE, scale='row', display_numbers=FALSE, display_genes=FALSE)
```

**SCATTERPLOT**: relationship between module membership and intramodular connectivity for up to 500 TURQUOISE genes (ranked according to connectivity).

```
plotGeneMetrics(GeneMetrics, module=ModName, title=NULL, top=500)
## Warning: Removed 14 rows containing missing values
## (geom_interactive_point).
```

**Interactive table** to explore genes assigned to TURQUOISE module

```
searchURL <- 'https://www.genecards.org/cgi-bin/carddisp.pl?gene='
# First part of the URL that will be used to generate the link

GeneMetrics %>% 
  dplyr::filter(Module==ModName) %>%
  dplyr::mutate(GeneLink=paste0('<a href="', searchURL, Gene, '">', Gene, '</a>')) %>% 
  # generation of the link
  dplyr::select(GeneLink, Module, hgnc_symbol, gene_biotype, kWithin, !!sym(paste0('MM', ModName)), !!sym(paste0('p_MM', ModName))) %>% 
  # selection of columns to be shown
  datatable(class='hover', rownames=FALSE, caption=paste(ModName, 'genes'), filter='top', options=list(pageLength=10, autoWidth=TRUE), escape=FALSE) %>%
  formatRound(c(5,6,7), c(2,2,6))
```

---

## 7. Pink Module

```
ModName <- 'pink'
```

PINK module is composed by 310 genes.

**BOXPLOT**: distribution of PINK module membership stratified for module assignment.

```
boxMM (GeneMM=GeneMetrics, module=ModName, title=NULL)
```

**RIBBON of Module Eigengene**: behavior of module eigengene through time-points and samples.

```
ribbonME(METraits=METraits, module=ModName, ascisse='Age', title=NULL)
```

**HEATMAP**: gene expression values (log transformed read counts) for the genes belonging to the PINK module.

```
heatmapGeneModule(data=LogMatrixSel, module=ModName, assignment=ModuleAssignment, title=NULL, annotation_col=HeatAnno, annotation_colors=HeatCols, annotation_row=NA, cluster_rows=TRUE, cluster_cols=TRUE, scale='row', display_numbers=FALSE, display_genes=FALSE)
```

**SCATTERPLOT**: relationship between module membership and intramodular connectivity for up to 500 PINK genes (ranked according to connectivity).

```
plotGeneMetrics(GeneMetrics, module=ModName, title=NULL, top=500)
## Warning: Removed 27 rows containing missing values
## (geom_interactive_point).
```

**Interactive table** to explore genes assigned to PINK module

```
GeneMetrics %>% 
  dplyr::filter(Module==ModName) %>%
  dplyr::mutate(GeneLink=paste0('<a href="', searchURL, Gene, '">', Gene, '</a>')) %>% 
  # generation of the link
  dplyr::select(GeneLink, Module, hgnc_symbol, gene_biotype, kWithin, !!sym(paste0('MM', ModName)), !!sym(paste0('p_MM', ModName))) %>% 
  # selection of columns to be shown
  datatable(class='hover', rownames=FALSE, caption=paste(ModName, 'genes'), filter='top', options=list(pageLength=10, autoWidth=TRUE), escape=FALSE) %>%
  formatRound(c(5,6,7), c(2,2,6))
```

---

## 8. Grey60 Module

```
ModName <- 'grey60'
```

GREY60 module is composed by 64 genes.

**BOXPLOT**: distribution of GREY60 module membership stratified for module assignment.

```
boxMM (GeneMM=GeneMetrics, module=ModName, title=NULL)
```

**RIBBON of Module Eigengene**: behavior of module eigengene through time-points and samples.

```
ribbonME(METraits=METraits, module=ModName, ascisse='Age', title=NULL)
```

**HEATMAP**: gene expression values (log transformed read counts) for the genes belonging to the GREY60 module.

```
heatmapGeneModule(data=LogMatrixSel, module=ModName, assignment=ModuleAssignment, title=NULL, annotation_col=HeatAnno, annotation_colors=HeatCols, annotation_row=NA, cluster_rows=TRUE, cluster_cols=TRUE, scale='row', display_numbers=FALSE, display_genes=FALSE)
```

**SCATTERPLOT**: relationship between module membership and intramodular connectivity for up to 500 GREY60 genes (ranked according to connectivity).

```
plotGeneMetrics(GeneMetrics, module=ModName, title=NULL, top=500)
## Warning: Removed 4 rows containing missing values (geom_interactive_point).
```

**Interactive table** to explore genes assigned to GREY60 module

```
GeneMetrics %>% 
  dplyr::filter(Module==ModName) %>%
  dplyr::mutate(GeneLink=paste0('<a href="', searchURL, Gene, '">', Gene, '</a>')) %>% 
  # generation of the link
  dplyr::select(GeneLink, Module, hgnc_symbol, gene_biotype, kWithin, !!sym(paste0('MM', ModName)), !!sym(paste0('p_MM', ModName))) %>% 
  # selection of columns to be shown
  datatable(class='hover', rownames=FALSE, caption=paste(ModName, 'genes'), filter='top', options=list(pageLength=10, autoWidth=TRUE), escape=FALSE) %>%
  formatRound(c(5,6,7), c(2,2,6))
```

---

## 9. MidnightBlue Module

```
ModName <- 'midnightblue'
```

MIDNIGHTBLUE module is composed by 105 genes.

**BOXPLOT**: distribution of MIDNIGHTBLUE module membership stratified for module assignment.

```
boxMM (GeneMM=GeneMetrics, module=ModName, title=NULL)
```

**RIBBON of Module Eigengene**: behavior of module eigengene through time-points and samples.

```
ribbonME(METraits=METraits, module=ModName, ascisse='Age', title=NULL)
```

**HEATMAP**: gene expression values (log transformed read counts) for the genes belonging to the MIDNIGHTBLUE module.

```
heatmapGeneModule(data=LogMatrixSel, module=ModName, assignment=ModuleAssignment, title=NULL, annotation_col=HeatAnno, annotation_colors=HeatCols, annotation_row=NA, cluster_rows=TRUE, cluster_cols=TRUE, scale='row', display_numbers=FALSE, display_genes=FALSE)
```

**SCATTERPLOT**: relationship between module membership and intramodular connectivity for up to 500 MIDNIGHTBLUE genes (ranked according to connectivity).

```
plotGeneMetrics(GeneMetrics, module=ModName, title=NULL, top=500)
## Warning: Removed 5 rows containing missing values (geom_interactive_point).
```

**Interactive table** to explore genes assigned to MIDNIGHTBLUE module

```
GeneMetrics %>% 
  dplyr::filter(Module==ModName) %>%
  dplyr::mutate(GeneLink=paste0('<a href="', searchURL, Gene, '">', Gene, '</a>')) %>% 
  # generation of the link
  dplyr::select(GeneLink, Module, hgnc_symbol, gene_biotype, kWithin, !!sym(paste0('MM', ModName)), !!sym(paste0('p_MM', ModName))) %>% 
  # selection of columns to be shown
  datatable(class='hover', rownames=FALSE, caption=paste(ModName, 'genes'), filter='top', options=list(pageLength=10, autoWidth=TRUE), escape=FALSE) %>%
  formatRound(c(5,6,7), c(2,2,6))
```

---

## 10. Black Module

```
ModName <- 'black'
```

BLACK module is composed by 343 genes.

**BOXPLOT**: distribution of BLACK module membership stratified for module assignment.

```
boxMM (GeneMM=GeneMetrics, module=ModName, title=NULL)
```

**RIBBON of Module Eigengene**: behavior of module eigengene through time-points and samples.

```
ribbonME(METraits=METraits, module=ModName, ascisse='Age', title=NULL)
```

**HEATMAP**: gene expression values (log transformed read counts) for the genes belonging to the BLACK module.

```
heatmapGeneModule(data=LogMatrixSel, module=ModName, assignment=ModuleAssignment, title=NULL, annotation_col=HeatAnno, annotation_colors=HeatCols, annotation_row=NA, cluster_rows=TRUE, cluster_cols=TRUE, scale='row', display_numbers=FALSE, display_genes=FALSE)
```

**SCATTERPLOT**: relationship between module membership and intramodular connectivity for up to 500 BLACK genes (ranked according to connectivity).

```
plotGeneMetrics(GeneMetrics, module=ModName, title=NULL, top=500)
## Warning: Removed 8 rows containing missing values (geom_interactive_point).
```

**Interactive table** to explore genes assigned to BLACK module

```
GeneMetrics %>% 
  dplyr::filter(Module==ModName) %>%
  dplyr::mutate(GeneLink=paste0('<a href="', searchURL, Gene, '">', Gene, '</a>')) %>% 
  # generation of the link
  dplyr::select(GeneLink, Module, hgnc_symbol, gene_biotype, kWithin, !!sym(paste0('MM', ModName)), !!sym(paste0('p_MM', ModName))) %>% 
  # selection of columns to be shown
  datatable(class='hover', rownames=FALSE, caption=paste(ModName, 'genes'), filter='top', options=list(pageLength=10, autoWidth=TRUE), escape=FALSE) %>%
  formatRound(c(5,6,7), c(2,2,6))
```

---

## 11. Yellow Module

```
ModName <- 'yellow'
```

YELLOW module is composed by 476 genes.

**BOXPLOT**: I visualize the distribution of YELLOW module membership stratified for module assignment.

```
boxMM (GeneMM=GeneMetrics, module=ModName, title=NULL)
```

**RIBBON of Module Eigengene**:behavior of module eigengene through time-points and samples.

```
ribbonME(METraits=METraits, module=ModName, ascisse='Age', title=NULL)
```

**HEATMAP**: gene expression values (log transformed read counts) for the genes belonging to the YELLOW module.

```
heatmapGeneModule(data=LogMatrixSel, module=ModName, assignment=ModuleAssignment, title=NULL, annotation_col=HeatAnno, annotation_colors=HeatCols, annotation_row=NA, cluster_rows=TRUE, cluster_cols=TRUE, scale='row', display_numbers=FALSE, display_genes=FALSE)
```

**SCATTERPLOT**: relationship between module membership and intramodular connectivity for up to 500 YELLOW genes (ranked according to connectivity).

```
plotGeneMetrics(GeneMetrics, module=ModName, title=NULL, top=500)
## Warning: Removed 20 rows containing missing values
## (geom_interactive_point).
```

**Interactive table** to explore genes assigned to YELLOW module

```
GeneMetrics %>% 
  dplyr::filter(Module==ModName) %>%
  dplyr::mutate(GeneLink=paste0('<a href="', searchURL, Gene, '">', Gene, '</a>')) %>% 
  # generation of the link
  dplyr::select(GeneLink, Module, hgnc_symbol, gene_biotype, kWithin, !!sym(paste0('MM', ModName)), !!sym(paste0('p_MM', ModName))) %>% 
  # selection of columns to be shown
  datatable(class='hover', rownames=FALSE, caption=paste(ModName, 'genes'), filter='top', options=list(pageLength=10, autoWidth=TRUE), escape=FALSE) %>%
  formatRound(c(5,6,7), c(2,2,6))
```

---

## 12. Blue Module

```
ModName <- 'blue'
```

BLUE module is composed by 776 genes.

**BOXPLOT**: visualize the distribution of BLUE module membership stratified for module assignment.

```
boxMM (GeneMM=GeneMetrics, module=ModName, title=NULL)
```

**RIBBON of Module Eigengene**: behavior of module eigengene through time-points and samples.

```
ribbonME(METraits=METraits, module=ModName, ascisse='Age', title=NULL)
```

**HEATMAP**: gene expression values (log transformed read counts) for the genes belonging to the BLUE module.

```
heatmapGeneModule(data=LogMatrixSel, module=ModName, assignment=ModuleAssignment, title=NULL, annotation_col=HeatAnno, annotation_colors=HeatCols, annotation_row=NA, cluster_rows=TRUE, cluster_cols=TRUE, scale='row', display_numbers=FALSE, display_genes=FALSE)
```

**SCATTERPLOT**:relationship between module membership and intramodular connectivity for up to 500 BLUE genes (ranked according to connectivity).

```
plotGeneMetrics(GeneMetrics, module=ModName, title=NULL, top=500)
## Warning: Removed 10 rows containing missing values
## (geom_interactive_point).
```

**Interactive table** explore genes assigned to BLUE module

```
GeneMetrics %>% 
  dplyr::filter(Module==ModName) %>%
  dplyr::mutate(GeneLink=paste0('<a href="', searchURL, Gene, '">', Gene, '</a>')) %>% 
  # generation of the link
  dplyr::select(GeneLink, Module, hgnc_symbol, gene_biotype, kWithin, !!sym(paste0('MM', ModName)), !!sym(paste0('p_MM', ModName))) %>% 
  # selection of columns to be shown
  datatable(class='hover', rownames=FALSE, caption=paste(ModName, 'genes'), filter='top', options=list(pageLength=10, autoWidth=TRUE), escape=FALSE) %>%
  formatRound(c(5,6,7), c(2,2,6))
```

---

#### 13. Red Module

```
ModName <- 'red'
```

RED module is composed by 375 genes.

**BOXPLOT**: distribution of RED module membership stratified for module assignment.

```
boxMM (GeneMM=GeneMetrics, module=ModName, title=NULL)
```

**RIBBON of Module Eigengene**: behavior of module eigengene through time-points and samples.

```
ribbonME(METraits=METraits, module=ModName, ascisse='Age', title=NULL)
```

**HEATMAP**: gene expression values (log transformed read counts) for the genes belonging to the RED module.

```
heatmapGeneModule(data=LogMatrixSel, module=ModName, assignment=ModuleAssignment, title=NULL, annotation_col=HeatAnno, annotation_colors=HeatCols, annotation_row=NA, cluster_rows=TRUE, cluster_cols=TRUE, scale='row', display_numbers=FALSE, display_genes=FALSE)
```

**SCATTERPLOT**: relationship between module membership and intramodular connectivity for up to 500 RED genes (ranked according to connectivity).

```
plotGeneMetrics(GeneMetrics, module=ModName, title=NULL, top=500)
## Warning: Removed 7 rows containing missing values (geom_interactive_point).
```

**Interactive table** to explore genes assigned to RED module

```
GeneMetrics %>% 
  dplyr::filter(Module==ModName) %>%
  dplyr::mutate(GeneLink=paste0('<a href="', searchURL, Gene, '">', Gene, '</a>')) %>% 
  # generation of the link
  dplyr::select(GeneLink, Module, hgnc_symbol, gene_biotype, kWithin, !!sym(paste0('MM', ModName)), !!sym(paste0('p_MM', ModName))) %>% 
  # selection of columns to be shown
  datatable(class='hover', rownames=FALSE, caption=paste(ModName, 'genes'), filter='top', options=list(pageLength=10, autoWidth=TRUE), escape=FALSE) %>%
  formatRound(c(5,6,7), c(2,2,6))
```

---

## 14. Session Info

```
date()
## [1] "Tue Jul 13 18:25:04 2021"

sessionInfo()
## R version 3.4.4 (2018-03-15)
## Platform: x86_64-pc-linux-gnu (64-bit)
## Running under: Ubuntu 18.04.2 LTS
## 
## Matrix products: default
## BLAS: /usr/lib/x86_64-linux-gnu/openblas/libblas.so.3
## LAPACK: /usr/lib/x86_64-linux-gnu/libopenblasp-r0.2.20.so
## 
## locale:
##  [1] LC_CTYPE=en_US.UTF-8       LC_NUMERIC=C              
##  [3] LC_TIME=en_US.UTF-8        LC_COLLATE=en_US.UTF-8    
##  [5] LC_MONETARY=en_US.UTF-8    LC_MESSAGES=en_US.UTF-8   
##  [7] LC_PAPER=en_US.UTF-8       LC_NAME=C                 
##  [9] LC_ADDRESS=C               LC_TELEPHONE=C            
## [11] LC_MEASUREMENT=en_US.UTF-8 LC_IDENTIFICATION=C       
## 
## attached base packages:
## [1] stats     graphics  grDevices utils     datasets  methods   base     
## 
## other attached packages:
##  [1] gdtools_0.1.7         WGCNA_1.64-1          fastcluster_1.1.25   
##  [4] dynamicTreeCut_1.63-1 ggiraph_0.5.0         ggplot2_3.1.0        
##  [7] dplyr_0.8.3           tidyr_0.8.1           DT_0.4               
## [10] pheatmap_1.0.10       viridis_0.5.1         viridisLite_0.3.0    
## 
## loaded via a namespace (and not attached):
##  [1] matrixStats_0.54.0    fit.models_0.5-14     robust_0.4-18        
##  [4] bit64_0.9-7           doParallel_1.0.14     RColorBrewer_1.1-2   
##  [7] rprojroot_1.3-2       tools_3.4.4           backports_1.1.2      
## [10] R6_2.3.0              rpart_4.1-13          Hmisc_4.1-1          
## [13] DBI_1.0.0             lazyeval_0.2.1        BiocGenerics_0.24.0  
## [16] colorspace_1.3-2      nnet_7.3-12           withr_2.1.2.9000     
## [19] tidyselect_0.2.5      gridExtra_2.3         bit_1.1-14           
## [22] compiler_3.4.4        preprocessCore_1.40.0 Biobase_2.38.0       
## [25] htmlTable_1.12        xml2_1.2.0            officer_0.3.2        
## [28] labeling_0.3          scales_1.0.0          checkmate_1.8.5      
## [31] mvtnorm_1.0-8         DEoptimR_1.0-8        robustbase_0.93-3    
## [34] stringr_1.3.1         digest_0.6.18         foreign_0.8-71       
## [37] rmarkdown_1.10        rrcov_1.4-4           base64enc_0.1-3      
## [40] pkgconfig_2.0.2       htmltools_0.3.6       rvg_0.1.9            
## [43] htmlwidgets_1.3       rlang_0.4.2           rstudioapi_0.8       
## [46] RSQLite_2.1.1         impute_1.52.0         shiny_1.1.0          
## [49] jsonlite_1.5          crosstalk_1.0.0       acepack_1.4.1        
## [52] zip_1.0.0             magrittr_1.5          GO.db_3.5.0          
## [55] Formula_1.2-3         Matrix_1.2-17         Rcpp_1.0.3           
## [58] munsell_0.5.0         S4Vectors_0.16.0      stringi_1.2.4        
## [61] yaml_2.2.0            MASS_7.3-50           plyr_1.8.4           
## [64] grid_3.4.4            blob_1.1.1            promises_1.0.1       
## [67] parallel_3.4.4        crayon_1.3.4          lattice_0.20-35      
## [70] splines_3.4.4         knitr_1.20            pillar_1.4.2         
## [73] uuid_0.1-2            codetools_0.2-15      stats4_3.4.4         
## [76] glue_1.3.0            evaluate_0.11         latticeExtra_0.6-28  
## [79] data.table_1.11.8     httpuv_1.4.5          foreach_1.4.4        
## [82] gtable_0.2.0          purrr_0.3.3           assertthat_0.2.0     
## [85] mime_0.6              xtable_1.8-3          later_0.7.5          
## [88] pcaPP_1.9-73          survival_2.42-6       tibble_2.1.3         
## [91] iterators_1.0.10      AnnotationDbi_1.40.0  memoise_1.1.0        
## [94] IRanges_2.12.0        cluster_2.0.7-1
```

---

## Conclusions

- **Turquoise**: **782 genes**, among which 18.93 % of non-coding genes. It shows the strongest positive correlation with Age; module eigengene increases till PCW37, with steepest increase between 13 and 21. Among the genes: GABA receptors, glutamate receptors, GAD1, GAD2, LGI1 LGI4 (Schwann cells), some RAB proteins
- **Pink**: **310 genes**, among which 20.65 % of non-coding genes. It shows strong positive correlation with Age; module eigengene overall increases till PCW37, although with some fluctuations. Among the genes: several K channels.
- **Grey60**: **64 genes**, among which 17.19 % of non-coding genes. It shows strong positive correlation with Age; module eigengene increases till PCW17, than stabilizes. Among the genes: several calcium channels, DLGAP2.
- **MidnightBlue**: **105 genes**, among which 21.9 % of non-coding genes. It shows strong positive correlation with Age; module eigengene increases till PCW17, than fluctuate and slightly decreases at later stages. Among the genes: SATB2, CUX2, POU3F1.
- **Black**: **343 genes**, among which 48.98 % of non-coding genes. It shows strong negative correlation with Age; module eigengene steeply till PCW19, then stabilizes.
- **Yellow**: **476 genes**, among which 24.16 % of non-coding genes. It does not shows a strong negative correlation with Age; module eigengene steeply decrease from PCW9 to PCW12. Several KIF and CDC, MKI67, NEUROD4, NEUROG1, NEUROG2, EOMES.
- **Blue**: **776 genes**, among which 20.49 % of non-coding genes. It shows an overall negative correlation with Age; module eigengene strongly decrease from PCW 9 to 12, bu then increase again at 37.
- **Red**: **375 genes**, among which 7.47 % of non-coding genes. Module eigengene is quite variable, with a clear increase at PCW 37. Among the genes: a lot of collagen genes, BGN,
