# Supplementary material for: Benchmarking brain organoid recapitulation of fetal corticogenesis
Source: Transl Psychiatry. 2022 Dec 20;12:520. doi: 10.1038/s41398-022-02279-0 (PMC9767930; doi:10.1038/s41398-022-02279-0)
Supplement: Supplementary file 6 — Supplementary file 2 [file 41398_2022_2279_MOESM6_ESM.html]

WGCNA on Cortical Brain Organoids: Module Characterization


Code 

- Show All Code
- Hide All Code

# WGCNA on Cortical Brain Organoids: Module Characterization

**WGCNA analysis on in-house differentiated Cortical Brain Organoids (from day 25 to day 200): module characterization.**

## 1. SetUp

```
library(viridis)
## Loading required package: viridisLite
library(pheatmap)
library(DT)
library(tidyr)
library(dplyr)
## 
## Attaching package: 'dplyr'
## The following objects are masked from 'package:stats':
## 
##     filter, lag
## The following objects are masked from 'package:base':
## 
##     intersect, setdiff, setequal, union
library(ggplot2)
library(ggiraph)
library(WGCNA)
## Loading required package: dynamicTreeCut
## Loading required package: fastcluster
## 
## Attaching package: 'fastcluster'
## The following object is masked from 'package:stats':
## 
##     hclust
## 
## ==========================================================================
## *
## *  Package WGCNA 1.64.1 loaded.
## *
## *    Important note: It appears that your system supports multi-threading,
## *    but it is not enabled within WGCNA in R. 
## *    To allow multi-threading within WGCNA with all available cores, use 
## *
## *          allowWGCNAThreads()
## *
## *    within R. Use disableWGCNAThreads() to disable threading if necessary.
## *    Alternatively, set the following environment variable on your system:
## *
## *          ALLOW_WGCNA_THREADS=<number_of_processors>
## *
## *    for example 
## *
## *          ALLOW_WGCNA_THREADS=40
## *
## *    To set the environment variable in linux bash shell, type 
## *
## *           export ALLOW_WGCNA_THREADS=40
## *
## *     before running R. Other operating systems or shells will
## *     have a similar command to achieve the same aim.
## *
## ==========================================================================
## 
## Attaching package: 'WGCNA'
## The following object is masked from 'package:stats':
## 
##     cor
```

```
options(stringsAsFactors = FALSE)
```

```
source('../WGCNAHelper.R')
```

---

## 2. Data Upload

### 2.1 Gene Metadata

```
GeneInfo <- readRDS('/media/data/Organoids/NeurodevDatasets/4_DataExploration/AnnotationGenecode/Output/OutputAnnotation.rds')
GeneMeta <- GeneInfo$AnnotationDF %>% dplyr::select(1:5)
dim(GeneMeta)
## [1] 58288     5
```

### 2.2 Objects generated from the first step

```
load('/media/data/Organoids/NeurodevDatasets/6_WGCNA/Organoids/1_NetworkGeneration/NetworkGeneration.RData')
```

- adjacency: adjacency matrix
- dissTOM: TOM distance matrix
- DummyTraits: phenotypic traits
- ModuleAssignment: assignment for each gene to a module
- MEs: module eigengenes
- VstSel: selected, transformed, transposed expression matrix

### 2.3 Heatmap Annotation

```
HeatAnno <- readRDS('/media/data/Organoids/NeurodevDatasets/4B_MergedDataExploration/InternalOrganoid/ExplorationInput/HeatmapAnno/HeatAnnoOrgWtSebaAll.rds') %>% dplyr::select(1,2,4)
HeatCols <- readRDS('/media/data/Organoids/NeurodevDatasets/4B_MergedDataExploration/InternalOrganoid/ExplorationInput/HeatmapAnno/HeatColorsOrgWtSebaAll.rds')
```

### 2.4 Sample nomenclature

- CTL1: A15461
- CTL2: KOLF2C1
- CTL3: MIFF1
- CTL4: 3391B

---

## 3. Module correlation with phenotypic traits

### 3.1 Correlation between module eigengenes and phenotypic traits

Add to the dummy variables also the information about sample identity.

```
DummyTraits$sample <- factor(c(rep('Kolf2C1', 6), rep('Miff1', 6), rep('3391B', 6), 
            rep('A15461', 6), rep(c('A15461', 'Kolf2C1', '3391B', 'Miff1'), 3), 
            'Kolf2C1', '3391B', 'Miff1'))

for(level in unique(factor(DummyTraits$sample))){
  DummyTraits[paste("Sample", level, sep = "_")] <- ifelse(DummyTraits$sample == level, 1, 0)
}

DummyTraits <- DummyTraits[, c(1:8, 10:13)]
```

Calculate the correlation with modules and the phenotypic traits using spearman correlation.

```
nGenes <- ncol(VstSel) 
nSamples <- nrow(VstSel)

moduleTraitCor <- WGCNA::cor(MEs, DummyTraits, use = 'p', method='spearman')
moduleTraitPvalue <- WGCNA::corPvalueStudent(moduleTraitCor, nSamples)
```

### 3.2 Visualization of the correlation

**Complete heatmap**

```
textMatrix <- paste(signif(moduleTraitCor,2), '\n(', signif(moduleTraitPvalue, 1), ')', sep = '')
dim(textMatrix) <- dim(moduleTraitCor)
WGCNA::labeledHeatmap(Matrix=moduleTraitCor, xLabels=names(DummyTraits), yLabels=names(MEs), ySymbols=names(MEs), colorLabels=FALSE, colors=viridis(50)[10:50], textMatrix = textMatrix, setStdMargins = FALSE, cex.text = 0.5, zlim = c(-1,1), main = paste('Module-trait relationships'))
```

**Selected heatmap**

```
textMatrix <- paste(signif(moduleTraitCor[, 2:7],2), '\n(', signif(moduleTraitPvalue[, 2:7], 1), ')', sep = '')
dim(textMatrix) <- dim(moduleTraitCor[, 2:7])
WGCNA::labeledHeatmap(Matrix=moduleTraitCor[, 2:7], xLabels=names(DummyTraits[, 2:7]), yLabels=names(MEs), ySymbols=names(MEs), colorLabels=FALSE, colors=viridis(50)[10:50], textMatrix=textMatrix, setStdMargins = FALSE, cex.text = 0.5, zlim = c(-1,1), main = paste('Module-trait relationships'))
```

**Overall Positive correlation with developmental day:**

- **Turquoise**: highest positive correlation with day.
- **Black**: positive correlation with day. Increases till d100, than slightly decrease.

**Overall Negative correlation with developmental day:**

- **Brown**: highest negative correlation with day.
- **Blue**: very strong negative correlation with day. Correlation peaks at d25, decreases until d100, than stable.
- **Magenta**: very strong negative correlation with day. Positive correlation peaks at day 50, than decreases.
- **Green**: mild negative correlation with day. Positive peaks at d25, negative peaks at d100.

**No strong correlation with developmental day:**

- **Pink, Yellow**: negative d25, peak positive at d50, than decrease again.
- **Salmon**: peak of negativ correlation at d50, while positive at d25 and d100.
- **Red**: positive (significant) at day25, negative (significant) at day50.
- **Purple**: weak negative (barely significant) at day25.
- **Cyan, Tan, GreenYellow**: no clear behaviour.

**Correlation with sample identity:**

- Tan, Cyan and GreenYellow show a noticeable degree of correlation with some sample identities.

---

## 4. Gene-related metrics: Module Membership and Connectivity

### 4.1 Calculate Gene Module Membership

The **Gene Module Membership** is a metrics that quantify the memebrship of each gene to all the identified modules.

Calculation of the correlation coefficient:

```
# Calculation of correlation coefficient and p-value 
Cor <- corAndPvalue(VstSel, MEs, use='p',  method='spearman')

CorCoeff <- data.frame(Cor$cor)
names(CorCoeff) <- paste0('MM', substring(names(CorCoeff), 3))
CorCoeff$Gene <- row.names(Cor$cor)

CorP <- data.frame(Cor$p)
names(CorP) <- paste0('p_MM', substring(names(CorP), 3))
CorP$Gene <- row.names(Cor$p)

# Generation of data frame containing relevant information
GeneMM <- dplyr::inner_join(CorCoeff, CorP, by='Gene') %>% 
  dplyr::inner_join(ModuleAssignment, by='Gene') %>%
  dplyr::inner_join(GeneMeta, by='Gene') %>% dplyr::select(33, 32, 1:15, 17:31, 16, 34:36)

dim(GeneMM)
## [1] 7831   36
```

### 4.2 Connectivity Measures

**Intramodular connectivity (kWithin)** calculates for each gene the connectivity with other genes belonging to the same module. Other measures that are calculated are the total connectivity (kTotal), the connectivity outside the module (kOut) and the difference between intra-modular and extra-modular connectivity (kDiff). All the metrics calculated at the gene level (module membership and connectivity) are stored in the **GeneMetrics** data frame.

```
GeneCon <- intramodularConnectivity(adjacency, colors=ModuleAssignment$Module)
GeneCon$Gene <- row.names(GeneCon)
GeneMetrics <- dplyr::inner_join(GeneMM, GeneCon, by='Gene')
```

---

## 5. Turquoise Module

```
ModName <- 'turquoise'
```

TURQUOISE module is composed by 3279 genes.

**BOXPLOT**: visualization of the distribution of TURQUOISE module membership stratified for module assignment.

```
boxMM(GeneMM=GeneMetrics, module=ModName, title=NULL)
```

**RIBBON of Module Eigengene**: visualization of the behaviour of module eigengene through time-points and samples.

```
# I add information about the lines-replicates
Rep <- c(rep(c('CTL2', 'CTL2_2'), 3), rep(c('CTL3', 'CTL3_2'), 3), rep(c('CTL4', 'CTL4_2'), 3), 
         rep(c('CTL1', 'CTL1_2'), 3), 'CTL1', 'CTL2', 'CTL4', 'CTL3', 'CTL1_2', 'CTL2_2', 'CTL4_2', 'CTL3_2', 
         'CTL1', 'CTL2', 'CTL4', 'CTL3','CTL2_2', 'CTL4_2', 'CTL3_2')

METraits <- dplyr::mutate(MEs, Sample=row.names(VstSel)) %>% inner_join(mutate(DummyTraits, Sample=row.names(DummyTraits)), by='Sample') %>% 
  mutate(Rep=Rep)

ribbonME(METraits=METraits, module=ModName, ascisse='Day', title=NULL)
```

**HEATMAP**: visualization of gene expression values (variance-stabilized and log transformed read counts) for the genes belonging to the TURQUOISE module.

```
heatmapGeneModule(data=VstSel, module=ModName, assignment=ModuleAssignment, title=NULL, annotation_col=HeatAnno, annotation_colors=HeatCols, annotation_row=NA, cluster_rows=TRUE, cluster_cols=TRUE, scale='row', display_numbers=FALSE, display_genes=FALSE)
```

**SCATTERPLOT**: visualizes the relationship between module membership and intramodular connectivity for up to 500 TURQUOISE genes (ranked according to connectivity).

```
plotGeneMetrics(GeneMetrics, module=ModName, title=NULL, top=500)
```

**Interactive table** to explore genes assigned to TURQUOISE module

```
searchURL <- 'https://www.genecards.org/cgi-bin/carddisp.pl?gene='
# First part of the URL that will be used to generate the link

GeneMetrics %>% 
  dplyr::filter(Module==ModName) %>%
  dplyr::mutate(GeneLink=paste0('<a href="', searchURL, EnsGene, '">', EnsGene, '</a>')) %>% 
  # generation of the link
  dplyr::select(GeneLink, Module, hgnc_symbol, gene_biotype, kWithin, !!sym(paste0('MM', ModName)), !!sym(paste0('p_MM', ModName))) %>% 
  # selection of columns to be shown
  datatable(class='hover', rownames=FALSE, caption=paste(ModName, 'genes'), filter='top', options=list(pageLength=10, autoWidth=TRUE), escape=FALSE) %>%
  formatRound(c(5,6,7), c(2,2,6))
```

---

## 6. Black Module

```
ModName <- 'black'
```

BLACK module is composed by 180 genes.

**BOXPLOT**: distribution of BLACK module membership stratified for module assignment.

```
boxMM (GeneMM=GeneMetrics, module=ModName, title=NULL)
```

**RIBBON of Module Eigengene**: behaviour of module eigengene through time-points and samples.

```
ribbonME(METraits=METraits, module=ModName, ascisse='Day', title=NULL)
```

**HEATMAP**: gene expression values (variance-stabilized and log transformed read counts) for the genes beloning to the BLACK module.

```
heatmapGeneModule(data=VstSel, module=ModName, assignment=ModuleAssignment, title=NULL, annotation_col=HeatAnno, annotation_colors=HeatCols, annotation_row=NA, cluster_rows=TRUE, cluster_cols=TRUE, scale='row', display_numbers=FALSE, display_genes=FALSE)
```

**SCATTERPLOT**: relationship between module membership and intramodular connectivity for up to 500 BLACK genes (ranked according to connectivity).

```
plotGeneMetrics(GeneMetrics, module=ModName, title=NULL, top=500)
```

**Interactive table** to explore genes assigned to BLACK module

```
GeneMetrics %>% 
  dplyr::filter(Module==ModName) %>%
  dplyr::mutate(GeneLink=paste0('<a href="', searchURL, EnsGene, '">', EnsGene, '</a>')) %>% 
  # generation of the link
  dplyr::select(GeneLink, Module, hgnc_symbol, gene_biotype, kWithin, !!sym(paste0('MM', ModName)), !!sym(paste0('p_MM', ModName))) %>% 
  # selection of columns to be shown
  datatable(class='hover', rownames=FALSE, caption=paste(ModName, 'genes'), filter='top', options=list(pageLength=10, autoWidth=TRUE), escape=FALSE) %>%
  formatRound(c(5,6,7), c(2,2,6))
```

---

## 7. Brown Module

```
ModName <- 'brown'
```

BROWN module is composed by 933 genes.

**BOXPLOT**: distribution of BROWN module membership stratified for module assignment.

```
boxMM (GeneMM=GeneMetrics, module=ModName, title=NULL)
```

**RIBBON of Module Eigengene**: behaviour of module eigengene through time-points and samples.

```
ribbonME(METraits=METraits, module=ModName, ascisse='Day', title=NULL)
```

**HEATMAP**: gene expression values (variance-stabilized and log transformed read counts) for the genes beloning to the BROWN module.

```
heatmapGeneModule(data=VstSel, module=ModName, assignment=ModuleAssignment, title=NULL, annotation_col=HeatAnno, annotation_colors=HeatCols, annotation_row=NA, cluster_rows=TRUE, cluster_cols=TRUE, scale='row', display_numbers=FALSE, display_genes=FALSE)
```

**SCATTERPLOT**: relationship between module membership and intramodular connectivity for up to 500 BROWN genes (ranked according to connectivity).

```
plotGeneMetrics(GeneMetrics, module=ModName, title=NULL, top=500)
```

**Interactive table** to explore genes assigned to BROWN module

```
GeneMetrics %>% 
  dplyr::filter(Module==ModName) %>%
  dplyr::mutate(GeneLink=paste0('<a href="', searchURL, EnsGene, '">', EnsGene, '</a>')) %>% 
  # generation of the link
  dplyr::select(GeneLink, Module, hgnc_symbol, gene_biotype, kWithin, !!sym(paste0('MM', ModName)), !!sym(paste0('p_MM', ModName))) %>% 
  # selection of columns to be shown
  datatable(class='hover', rownames=FALSE, caption=paste(ModName, 'genes'), filter='top', options=list(pageLength=10, autoWidth=TRUE), escape=FALSE) %>%
  formatRound(c(5,6,7), c(2,2,6))
```

---

## 8. Blue Module

```
ModName <- 'blue'
```

BLUE module is composed by 1240 genes.

**BOXPLOT**: distribution of BLUE module membership stratified for module assignment.

```
boxMM (GeneMM=GeneMetrics, module=ModName, title=NULL)
```

**RIBBON of Module Eigengene**: behaviour of module eigengene through time-points and samples.

```
ribbonME(METraits=METraits, module=ModName, ascisse='Day', title=NULL)
```

**HEATMAP**: gene expression values (variance-stabilized and log transformed read counts) for the genes beloning to the BLUE module.

```
heatmapGeneModule(data=VstSel, module=ModName, assignment=ModuleAssignment, title=NULL, annotation_col=HeatAnno, annotation_colors=HeatCols, annotation_row=NA, cluster_rows=TRUE, cluster_cols=TRUE, scale='row', display_numbers=FALSE, display_genes=FALSE)
```

**SCATTERPLOT**: relationship between module membership and intramodular connectivity for up to 500 BLUE genes (ranked according to connectivity).

```
plotGeneMetrics(GeneMetrics, module=ModName, title=NULL, top=500)
```

**Interactive table** to explore genes assigned to BLUE module

```
GeneMetrics %>% 
  dplyr::filter(Module==ModName) %>%
  dplyr::mutate(GeneLink=paste0('<a href="', searchURL, EnsGene, '">', EnsGene, '</a>')) %>% 
  # generation of the link
  dplyr::select(GeneLink, Module, hgnc_symbol, gene_biotype, kWithin, !!sym(paste0('MM', ModName)), !!sym(paste0('p_MM', ModName))) %>% 
  # selection of columns to be shown
  datatable(class='hover', rownames=FALSE, caption=paste(ModName, 'genes'), filter='top', options=list(pageLength=10, autoWidth=TRUE), escape=FALSE) %>%
  formatRound(c(5,6,7), c(2,2,6))
```

---

## 9. Green Module

```
ModName <- 'green'
```

GREEN module is composed by 364 genes.

**BOXPLOT**: distribution of GREEN module membership stratified for module assignment.

```
boxMM (GeneMM=GeneMetrics, module=ModName, title=NULL)
```

**RIBBON of Module Eigengene**: behaviour of module eigengene through time-points and samples.

```
ribbonME(METraits=METraits, module=ModName, ascisse='Day', title=NULL)
```

**HEATMAP**: gene expression values (variance-stabilized and log transformed read counts) for the genes beloning to the GREEN module.

```
heatmapGeneModule(data=VstSel, module=ModName, assignment=ModuleAssignment, title=NULL, annotation_col=HeatAnno, annotation_colors=HeatCols, annotation_row=NA, cluster_rows=TRUE, cluster_cols=TRUE, scale='row', display_numbers=FALSE, display_genes=FALSE)
```

**SCATTERPLOT**: relationship between module membership and intramodular connectivity for up to 500 GREEN genes (ranked according to connectivity).

```
plotGeneMetrics(GeneMetrics, module=ModName, title=NULL, top=500)
```

**Interactive table** to explore genes assigned to GREEN module

```
GeneMetrics %>% 
  dplyr::filter(Module==ModName) %>%
  dplyr::mutate(GeneLink=paste0('<a href="', searchURL, EnsGene, '">', EnsGene, '</a>')) %>% 
  # generation of the link
  dplyr::select(GeneLink, Module, hgnc_symbol, gene_biotype, kWithin, !!sym(paste0('MM', ModName)), !!sym(paste0('p_MM', ModName))) %>% 
  # selection of columns to be shown
  datatable(class='hover', rownames=FALSE, caption=paste(ModName, 'genes'), filter='top', options=list(pageLength=10, autoWidth=TRUE), escape=FALSE) %>%
  formatRound(c(5,6,7), c(2,2,6))
```

---

## 10. Red Module

```
ModName <- 'red'
```

RED module is composed by 249 genes.

**BOXPLOT**: distribution of RED module membership stratified for module assignment.

```
boxMM (GeneMM=GeneMetrics, module=ModName, title=NULL)
```

**RIBBON of Module Eigengene**: behaviour of module eigengene through time-points and samples.

```
ribbonME(METraits=METraits, module=ModName, ascisse='Day', title=NULL)
```

**HEATMAP**: gene expression values (variance-stabilized and log transformed read counts) for the genes beloning to the RED module.

```
heatmapGeneModule(data=VstSel, module=ModName, assignment=ModuleAssignment, title=NULL, annotation_col=HeatAnno, annotation_colors=HeatCols, annotation_row=NA, cluster_rows=TRUE, cluster_cols=TRUE, scale='row', display_numbers=FALSE, display_genes=FALSE)
```

**SCATTERPLOT**: relationship between module membership and intramodular connectivity for up to 500 RED genes (ranked according to connectivity).

```
plotGeneMetrics(GeneMetrics, module=ModName, title=NULL, top=500)
```

**Interactive table** to explore genes assigned to RED module

```
GeneMetrics %>% 
  dplyr::filter(Module==ModName) %>%
  dplyr::mutate(GeneLink=paste0('<a href="', searchURL, EnsGene, '">', EnsGene, '</a>')) %>% 
  # generation of the link
  dplyr::select(GeneLink, Module, hgnc_symbol, gene_biotype, kWithin, !!sym(paste0('MM', ModName)), !!sym(paste0('p_MM', ModName))) %>% 
  # selection of columns to be shown
  datatable(class='hover', rownames=FALSE, caption=paste(ModName, 'genes'), filter='top', options=list(pageLength=10, autoWidth=TRUE), escape=FALSE) %>%
  formatRound(c(5,6,7), c(2,2,6))
```

---

## 11. Session Info

```
SessionInfo <- sessionInfo()
Date <- date()

SessionInfo <- sessionInfo()
```

---

## Conclusions

The following modules have been pinpointed as interesting:

- **Turquoise**: **3279 genes**, among which 4.76 % of non-coding genes. It shows the strongest positive correlation with Day; module eigengene increases till Day200.
- **Black**: **180 genes**, among which 8.89 % of non-coding genes. It shows a positive correlation with Day; module eigengene increases steeply between Day50 and Day100, then stabilizes. There is a degree of variability across samples, with 2 samples not showing any clear increase and another for which the increase continue till Day150.
- **Brown**: **933 genes**, among which 3.97 % of non-coding genes. It shows the strongest negative correlation with Day; module eigengene decrease from Day25 to Day200, with most steep decrease between day25 and day50 for most samples.
- **Blue**: **1240 genes**, among which 5.89 % of non-coding genes. It shows a very strong negative correlation with Day; module eigengene decrease steeply from Day25 to Day100, then it stabilizes.
- **Green**: **364 genes**, among which 1.37 % of non-coding genes. It shows a mild negative correlation with Day; module eigengene decreases steeply from Day25 to Day50, slightly to Day100 and then stabilizes or tend to increase.
- **Red**: **249 genes**, among which 1.61 % of non-coding genes. It shows no clear correlation with Day; module eigengene decreases steeply from Day25 to Day50, then tend to increase.
